# Interfacial Tandem Catalysis Over a CuSn(OH)_6_/Co Heterostructure Enables Efficient Electroreduction of Nitrate to Ammonia

**DOI:** 10.1002/advs.77640

**Published:** 2026-09-08

**Authors:** Yuan‐Yuan Hei, Qing Chen, Jie Gu, Yifan Leng, Yaping Huang, Sunhui Chen, Hong Sun, Hongfang Du

**Affiliations:** ^1^ Department of Pharmacy Fuzhou University Affiliated Provincial Hospital Fuzhou University Fuzhou China; ^2^ Strait Laboratory of Flexible Electronics (SLoFE) Strait Institute of Flexible Electronics (SIFE Future Technologies) Fujian Normal University Fuzhou China

**Keywords:** electrocatalysis, electrocatalyst, electrochemical synthesis of ammonia, heterostructure, nitrate reduction, selectivity, tandem catalysis

## Abstract

Electrochemical nitrate reduction reaction (NO_3_^−^RR) enables sustainable ammonia (NH_3_) production but is constrained by sluggish kinetics due to the high stability of NO_3_^−^ and insufficient hydrogenation of nitrogenous intermediates, where the initial NO_3_^−^ → NO_2_^−^ conversion is the rate‐determining step (RDS). Herein, we report a CuSn(OH)_6_/Co heterostructure designed via an interfacial tandem‐catalysis strategy to address both the kinetic bottleneck and downstream hydrogenation steps in a coordinated manner. In this architecture, CuSn(OH)_6_ activates NO_3_^−^ and then selectively catalyzes its conversion into *NO_2_, thereby overcoming the RDS limitation. Meanwhile, Co nanoparticles promote rapid H_2_O dissociation to generate abundant *H for subsequent hydrogenation reactions. Further investigations reveal that the heterointerface enables efficient directional transfer of *NO_2_ intermediate from CuSn(OH)_6_ to the Co component, promoting its complete reduction to NH_3_. Benefiting from this spatially and functionally integrated division of catalytic roles, the CuSn(OH)_6_/Co heterostructure exhibits a high NH_3_ yield of 64.51 µmol h^−^^1^ mg_Cat_^−^^1^ and a selectivity of 92.28% at −0.7 V in 0.1 M NaSO_4_ containing 10 mM NaNO_3_, outperforming most reported catalysts. This work highlights a tandem‐catalysis strategy that synchronously regulates RDS and hydrogenation pathways for sustainable NH_3_ production via NO_3_^−^RR.

## Graphical abstract

Interfacial tandem catalysis is developed for efficient electrochemical reduction of NO_3_^−^ to NH_3_ over a CuSn(OH)_6_/Co heterostructure. CuSn(OH)_6_ accelerates the rate‐determining NO_3_^−^‐to‐*NO_2_ conversion, whereas Co promotes H_2_O dissociation to enhance *H supply for the rapid hydrogenation of *NO_2_ intermediate to NH_3_. The synergistic heterointerface enables high NH_3_ yield and selectivity, offering an effective catalyst for sustainable NH_3_ production via NO_3_^−^RR.

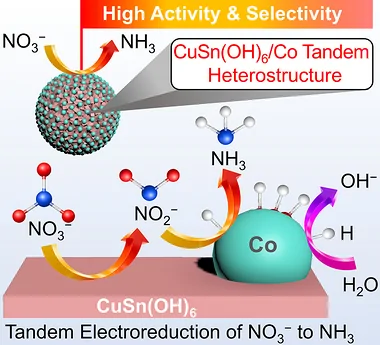

## 1 Introduction

Ammonia (NH_3_) is a cornerstone for global development, owing to its indispensable roles in nitrogen fertilizer synthesis, chemical manufacturing, and emerging potential as a carbon‐free hydrogen carrier, which together drive its ever‐increasing demand [1, 2, 3]. The sustainable production of NH_3_ has therefore attracted widespread attention in the context of carbon neutrality [4]. Among the emerging alternatives, the electrochemical nitrate reduction reaction (NO_3_^−^RR) has been recognized as a promising route to replace the energy‐ and carbon‐intensive Haber‐Bosch process, enabling NH_3_ synthesis under ambient conditions while simultaneously addressing nitrate pollution in wastewater [4, 5]. However, the NO_3_^−^RR involves a complex eight‐electron and nine‐proton transfer process, inevitably leading to sluggish reaction kinetics and diverse competing products such as NO_2_^−^, NO, N_2_, NH_2_OH, and H_2_ [6]. In particular, the sp^2^‐hybridized N atom binds strongly and equally to three O atoms with an O–N–O bond angle of 120° and an N–O bond length of 121 pm, resulting in a planar and symmetric configuration with a highly delocalized electronic distribution [3]. This endows NO_3_^−^ with high thermodynamic stability and significantly hinders initial activation [7]. Moreover, electrostatic repulsion between negatively charged NO_3_^−^ species and cathode surfaces further weakens interfacial adsorption and electron transfer [8]. As a result, the initial NO_3_^−^ → NO_2_^−^ conversion is generally regarded as the rate‐determining step (RDS) for NO_3_^−^RR [9, 10, 11].

To achieve efficient NO_3_^−^ to NH_3_ conversion, extensive research efforts have been dedicated to accelerating the RDS through catalyst design [11]. For example, nanostructure engineering can increase surface area to enlarge surface energy and expose abundant active sites, thereby enhancing NO_3_^−^ adsorption and facilitating *NO_3_ formation to promote RDS [12, 13]. While effective in improving apparent activity, such surface‐area‐dominated strategies often exhibit limited intrinsic activity enhancement and tend to be invalid once the physical adsorption is saturated. The electronic structure modulation via heteroatom doping, alloying, and defect engineering has been widely adopted to tune the adsorption strength of key intermediates and lower the activation barrier of RDS [14, 15]. However, these approaches typically assume a single‐site reaction pathway and therefore struggle to simultaneously optimize the multi‐step proton–electron transfer processes. Recent mechanistic insights suggest that improving the RDS alone is insufficient to achieve high NH_3_ selectivity [16]. After the initial NO_3_^−^ → NO_2_^−^ reaction, the inefficient NO_2_^−^ consumption leads to its accumulation and triggers competing reactions toward diverse byproducts, resulting in diminished NH_3_ selectivity [17]. The key is attributed to the insufficient supply of active hydrogen species (*H), arising from sluggish H_2_O dissociation kinetics [18, 19]. Taking both sides into account, a tandem catalysis strategy has thus been proposed to accelerate both NO_3_^−^ → NO_2_^−^ and NO_2_^−^ → NH_3_ reactions [20, 21, 22]. Nevertheless, the rational design and construction of tandem catalysts that can synchronize efficient NO_3_^−^ activation with rapid NO_2_^−^ hydrogenation remains highly challenging.

Perovskite hydroxide CuSn(OH)_6_ has been demonstrated to have high specific intrinsic activity toward the RDS NO_3_^−^ → NO_2_^−^ reaction in our recent work, owing to its unique compositional and structural configurations that enhance NO_3_^−^ adsorption and activation [23]. CuSn(OH)_6_ showed a high NO_2_^−^ yield of 148.5 µmol h^−^^1^ mg_Cat_^−^^1^ and a high selectivity of 97% at −0.7 V in 10 mM NaNO_3_ and 0.1 M Na_2_SO_4_ electrolyte, rendering it a promising candidate to construct high‐performance tandem catalysts. Herein, we report Co nanoparticles on CuSn(OH)_6_ microspheres, abbreviated as CuSn(OH)_6_/Co, as an interfacial tandem catalyst for highly efficient electrochemical reduction of NO_3_^−^ to NH_3_. In the CuSn(OH)_6_/Co heterostructure, CuSn(OH)_6_ can adsorb and activate NO_3_^−^ and facilitate its fast reduction to *NO_2_, thereby effectively accelerating RDS [23]. The *NO_2_ then migrates to nearby Co sites across the heterointerfaces. Concurrently, Co nanoparticles function as a hydrogenation domain, where efficient H_2_O dissociation generates abundant *H species and enables rapid hydrogenation of the *NO_2_ intermediate to NH_3_. Profiting from this spatially coordinated tandem catalytic mechanism, the CuSn(OH)_6_/Co catalyst achieves a high NH_3_ yield of 64.51 µmol h^−^^1^ mg_Cat_^−^^1^ with a high NH_3_ selectivity of 92.28% at −0.7 V in 0.1 M NaSO_4_ containing 10 mM NaNO_3_, substantially higher than both individual components and most reported catalysts. This work demonstrates an effective interfacial tandem‐catalysis strategy to regulate multi‐step reaction pathways and product selectivity in NO_3_^−^RR, offering new insights into the rational design of high‐performance electrocatalysts for sustainable NH_3_ electrosynthesis.

## 2 Results and Discussion

**FIGURE 1:**
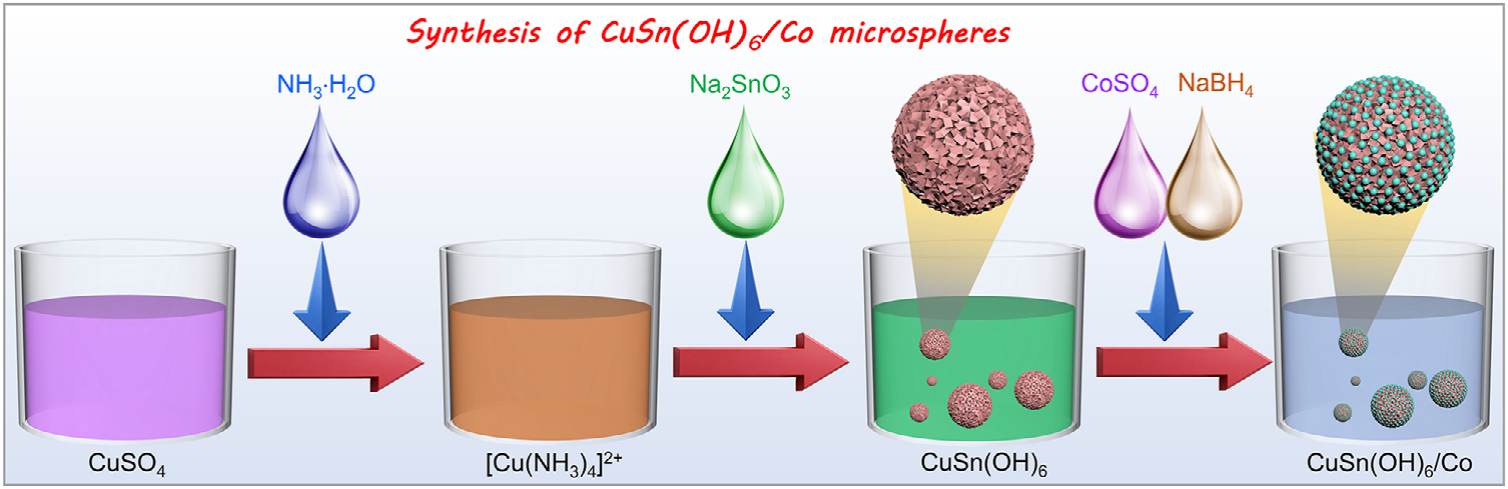
Schematic illustration for the synthesis of CuSn(OH)_6_/Co microspheres.

The CuSn(OH)_6_ microspheres were initially prepared as described in our previous report [23]. As schematically illustrated in Figure 1, a CuSO_4_ solution was mixed with excessive NH_3_∙H_2_O to form [Cu(NH_3_)_4_]^2+^ complex, which then reacted with Na_2_SnO_3_ solution to synthesize CuSn(OH)_6_ microspheres. The X‐ray diffraction (XRD) pattern of the resultant material exhibits clear diffraction peaks (Figure S1), all of which can be indexed to the perovskite hydroxide CuSn(OH)_6_ phase (JCPDS No. 70–0117) without detectable impurity peaks [24]. Scanning electron microscopy (SEM) and transmission electron microscopy (TEM) characterizations reveal that CuSn(OH)_6_ possesses a typical microspherical morphology (Figure S2a–c). Notably, the CuSn(OH)_6_ microsphere surface is assembled from densely packed nanocubes (Figure S2b,d), giving rise to a hierarchical architecture, consistent with previous reports [23]. In the high‐resolution TEM (HRTEM) image, distinct lattice fringes with an interplanar spacing of 0.38 nm are observed (Figure S2e), corresponding to the (200) facet of perovskite hydroxide CuSn(OH)_6_.

**FIGURE 2:**
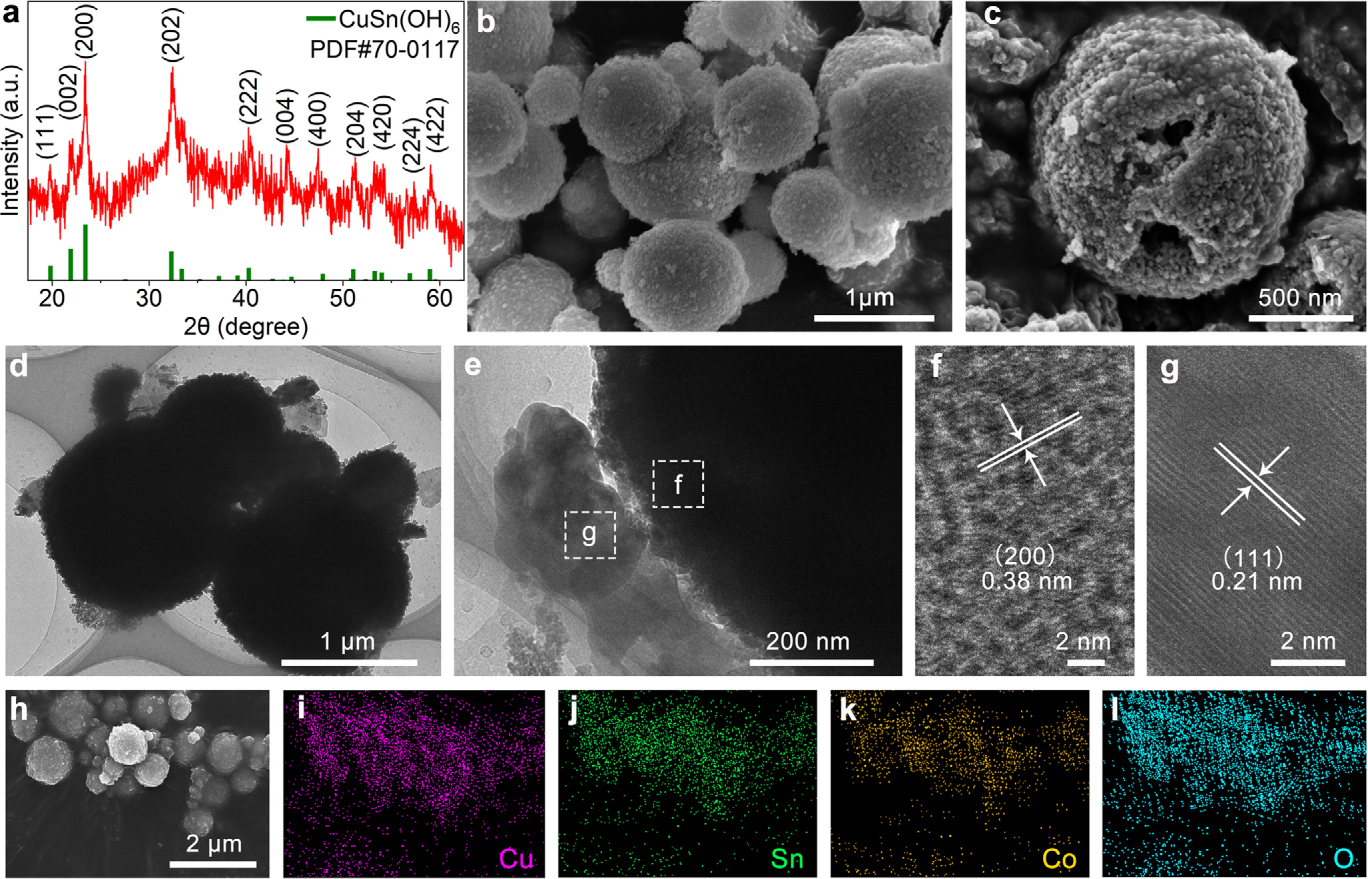
(a) XRD pattern of CuSn(OH)_6_/Co. (b, c) SEM, (d, e) TEM, and (f, g) HRTEM images of CuSn(OH)_6_/Co. (h) SEM and (i‐l) corresponding EDS mapping images of CuSn(OH)_6_/Co.

The as‐prepared CuSn(OH)_6_ microspheres were dispersed in aqueous CoSO_4_ solution, followed by chemical reduction with NaBH_4_ to construct CuSn(OH)_6_/Co heterostructures (Figure 1). The XRD pattern of CuSn(OH)_6_/Co synthesized at a CuSn(OH)_6_‐to‐Co molar ratio of 1:1 (i.e, a Co content of 50%) is highly similar to that of pristine CuSn(OH)_6_ (Figure 2a). No additional diffraction peaks associated with metallic Co are observed, suggesting that the chemically reduced Co nanoparticles possess poor crystallinity. The CuSn(OH)_6_/Co composite largely preserves the original microspherical morphology (Figure 2b–d). However, a remarkable change in surface texture is observed after Co incorporation. Specifically, the nanocube‐assembled surface of pristine CuSn(OH)_6_ is replaced by uniformly distributed smooth nanoparticles (Figure 2c,e). Such morphological evolution strongly indicates the successful incorporation of Co nanoparticles onto the CuSn(OH)_6_ surface. At the interface between a CuSn(OH)_6_ microsphere and an attached Co nanoparticle (Figure 2e), two sets of lattice fringes with spacings of 0.38 and 0.21 nm are clearly discerned (Figure 2f,g), corresponding to the (200) plane of CuSn(OH)_6_ and the (111) plane of metallic Co (JCPDS No. 71–4651), respectively. The contact between the two phases evidences the formation of CuSn(OH)_6_/Co heterointerfaces, which are expected to facilitate interfacial charge transfer during electrocatalysis [25, 26]. Furthermore, elemental mapping images reveal homogeneous distributions of Cu, Sn, Co, and O elements throughout the microspheres (Figure 2h,i), evidencing that Co nanoparticles are uniformly anchored on the CuSn(OH)_6_ framework without noticeable aggregation. Such uniform dispersion is advantageous for maximizing interfacial contact area and ensuring efficient utilization of active sites. The corresponding energy‐dispersive X‐ray spectroscopy (EDS) spectrum in Figure S3 further confirms the coexistence of Cu, Sn, Co, and O elements, and their relative contents are consistent with the stoichiometric ratio of CuSn(OH)_6_/Co (1:1). For comparison, Co nanoparticles were also synthesized by reducing CoSO_4_ with NaBH_4_ under identical conditions. The obtained sample exhibits no obvious diffraction peaks in the XRD pattern (Figure S1), confirming its low crystallinity. Further SEM and TEM analyses demonstrate a particulate nanostructure (Figure S4a–c). In the corresponding HRTEM image (Figure S4d), lattice fringes with a spacing of 0.21 nm are observed, corresponding to the (111) plane of metallic Co, confirming the successful synthesis of metallic Co nanoparticles.

**FIGURE 3:**
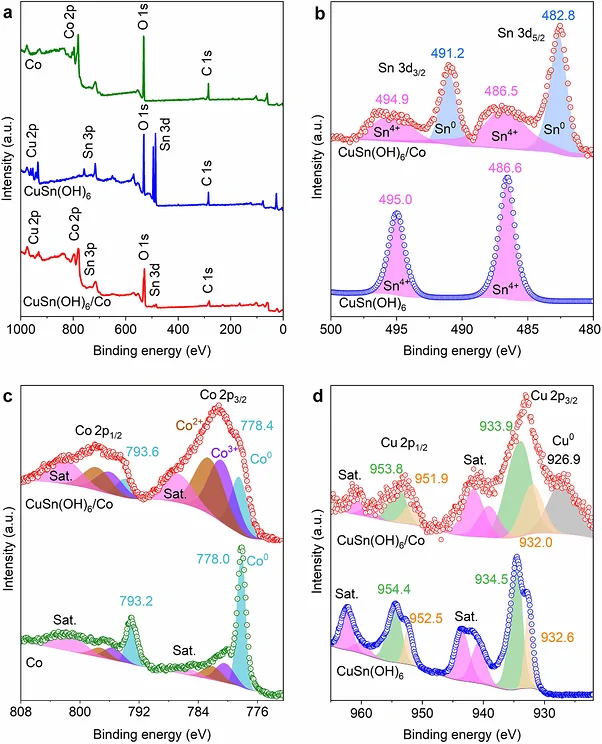
(a) Survey, (b) Sn 3d, (c) Cu 2p, and (d) Co 2p XPS spectra of CuSn(OH)_6_/Co, CuSn(OH)_6_, and Co nanoparticles.

To gain insight into the surface electronic structure and interfacial interactions, X‐ray photoelectron spectroscopy (XPS) measurements were performed. The survey XPS spectrum of CuSn(OH)_6_/Co (Figure 3a) again verifies the presence of Cu, Sn, Co, and O elements. The weak C 1s signal originates from adventitious carbon contamination commonly introduced during air exposure and sample handling. In contrast, the Co nanoparticles contain only Co and O elements, while no Co signal is detected for CuSn(OH)_6_ microspheres. In the Sn 3d region, pristine CuSn(OH)_6_ exhibits two characteristic peaks located at 486.6 and 495.0 eV (Figure 3b), corresponding to the Sn 3d_5/2_ and Sn 3d_3/2_ of Sn^4+^ species in the CuSn(OH)_6_ [23, 24]. As for CuSn(OH)_6_/Co, two pairs of Sn‐related peaks are observed. The pair of peaks at 486.5 and 494.9 eV is assigned to Sn^4+^ in the CuSn(OH)_6_ phase, whereas an additional pair of peaks at 482.8 and 491.2 eV is attributed to Sn^0^ species. The emergence of Sn^0^ indicates partial surface reduction of CuSn(OH)_6_ during NaBH_4_ treatment, suggesting that the reduction process induces localized surface reconstruction while maintaining the bulk crystal structure. The Co 2p spectrum of Co nanoparticles could be deconvoluted into three pairs of characteristic peaks accompanied by a couple of satellite (Sat.) peaks (Figure 3c). The pair of dominant peaks located at low binding energies of 778.0 and 793.2 eV is assignable to the Co 2p_3/2_ and Co 2p_1/2_ of Co^0^ [27]. The remaining weaker peaks at higher binding energies correspond to Co^3^^+^ and Co^2^^+^ species arising from superficial oxidation upon air exposure. Compared with Co nanoparticles, the Co^0^ peaks in CuSn(OH)_6_/Co positively shift by approximately 0.4 eV to 778.4 and 793.6 eV. Meanwhile, the relative intensities of Co^3^^+^ and Co^2^^+^ species increase dramatically, indicating a more electron‐deficient Co environment after heterostructure formation [28]. The Cu 2p spectrum of CuSn(OH)_6_ microspheres consists of two pairs of subpeaks together with Sat. peaks (Figure 3d). The peaks located at 934.5 and 954.4 eV are indexed to surface Cu hydro/oxide species, whereas those at 932.6 and 952.5 eV correspond to Cu^2^^+^ species within the CuSn(OH)_6_ lattice [23]. In the CuSn(OH)_6_/Co composite, the Cu‐related peaks associated with the CuSn(OH)_6_ phase shift negatively by approximately 0.6 eV. In addition, a Cu^0^ signal appears at 926.9 eV, which can be attributed to the partial reduction of surface Cu^2+^ species during NaBH_4_ treatment. Compared with individual components, the positive shift of Co 2p and the negative shift of Cu 2p in the CuSn(OH)_6_/Co heterostructure indicate electron transfer from metallic Co to the CuSn(OH)_6_ phase [28, 29]. This charge redistribution establishes a built‐in electric field across the heterointerface, which is expected to accelerate directional electron transport for fast electrode kinetics [30, 31].

**FIGURE 4:**
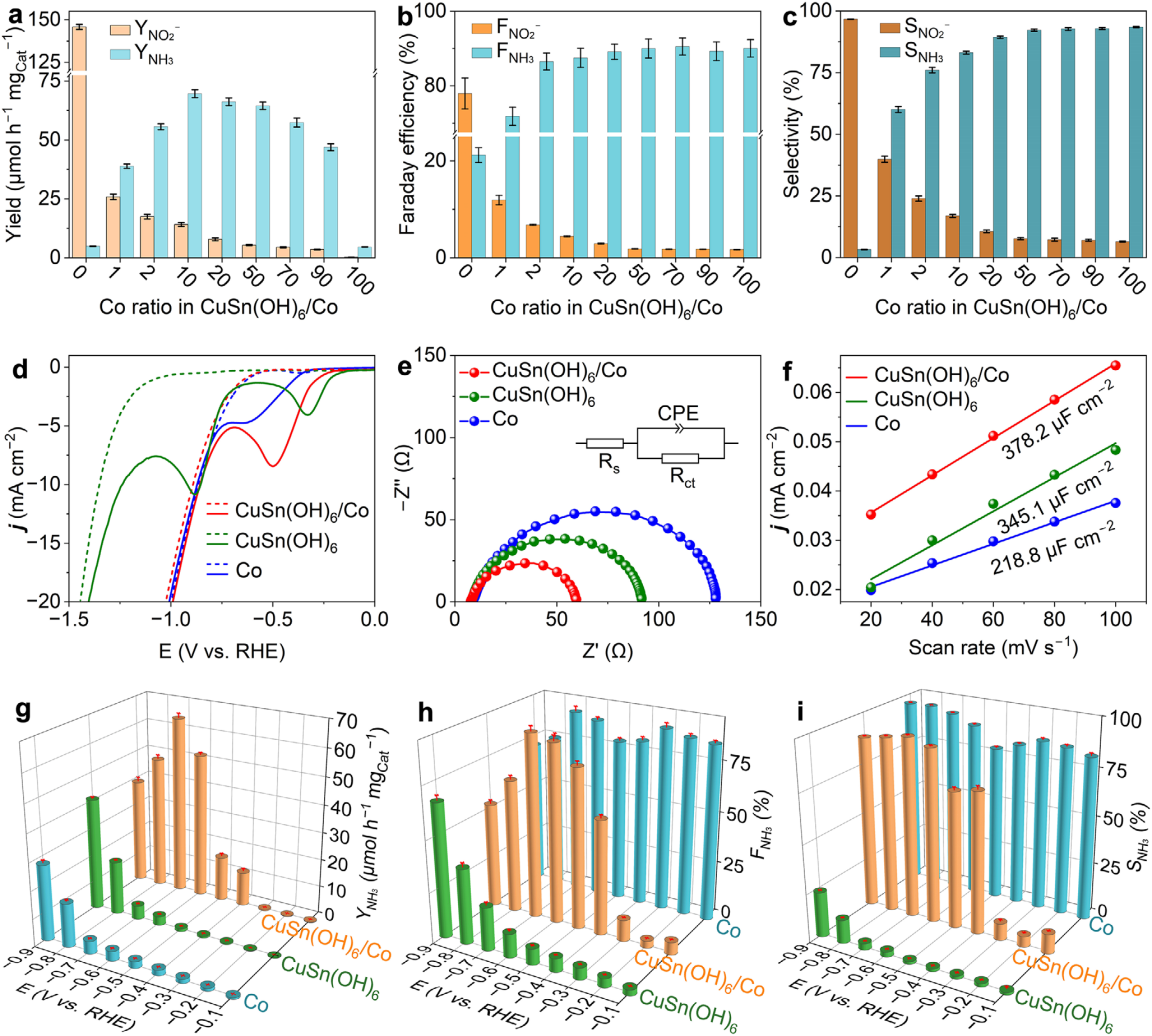
NH_4_^+^ and NO_2_^−^ products (a) yield, (b) Faraday efficiency, and (c) selectivity at −0.7 for CuSn(OH)_6_/Co catalysts synthesized at various Co contents. (d) Polarization curves, (e) Nyquist plots, and (f) double‐layer capacitance (C_dl_) of CuSn(OH)_6_/Co, CuSn(OH)_6_, and Co nanoparticles. (g) $Y_{NH_{3}},$ (h) $F_{NH_{3}},$ and (i) $S_{NH_{3}}$ of CuSn(OH)_6_/Co, CuSn(OH)_6_, and Co nanoparticles.

To better reflect the intrinsic electrochemical behavior, CuSn(OH)_6_/Co and associated catalysts were evaluated with a low‐concentration NaNO_3_ (10 mM) electrolyte containing 0.1 M Na_2_SO_4_ as a supporting electrolyte. After bulk electrolysis at −0.7 V for 30 min, the reduction products were quantified via spectrophotometry (Figures S5–S7). As shown in Figure S8, NH_3_ and NO_2_^−^ are identified as the major nitrogen‐containing reduction products in the electrolyte, whereas hydrazine (N_2_H_4_) is below the detection limit. Moreover, only a trace amount of H_2_ has been detected in the gas phase (Figure S9), without the detection of gaseous nitrogen‐containing products, such as N_2_, NO, and N_2_O. The detailed data sheets are presented in Tables S1 and S2. As shown in Figure 4a, the NH_3_ yield ($Y_{NH_{3}}$) of CuSn(OH)_6_/Co composite exhibits a volcano‐shaped dependence on the Co content, reaching a maximum value of 69.64 µmol h^−^^1^ mg_Cat_^−^^1^ at a Co content of 10%. In contrast, NO_2_^−^ yield ($Y_{{\mathrm{NO}}_{2}^{\;-}}$) gradually decreases with increasing Co content. The volcano‐type trend of $Y_{NH_{3}}$ suggests that an appropriate amount of Co is essential for establishing an effective tandem catalytic pathway. Insufficient Co loading cannot provide adequate hydrogenation capability, whereas excessive Co may overcover the NO_3_^−^ adsorption sites on CuSn(OH)_6_, thereby suppressing overall activity. Moreover, the NH_3_ Faraday efficiency ($F_{NH_{3}}$) of CuSn(OH)_6_/Co continuously increases with rising Co content (Figure 4b), whereas the NO_2_^−^ Faraday efficiency ($F_{{\mathrm{NO}}_{2}^{\;-}}$) displays an opposite tendency. Consistently, the NH_3_ selectivity ($S_{NH_{3}}$), defined as the relative ratio of NH_3_ to other nitrogen‐containing reduction products, of CuSn(OH)_6_/Co share simialr faturea to that $F_{NH_{3}}$ with the increase of Co ratio, and the NO_2_^−^ selectivity ($S_{{\mathrm{NO}}_{2}^{\;-}}$) trend resembles that of $F_{{\mathrm{NO}}_{2}^{\;-}}$ (Figure 4c). These results indicate that Co nanoparticles effectively promote the conversion of *NO_2_ intermediate into NH_3_, thereby suppressing intermediate accumulation and enhancing overall NH_3_ selectivity [32]. Although CuSn(OH)_6_/Co with 10% Co exhibits a higher $Y_{NH_{3}}$ (69.64 µmol h^−^^1^ mg^−^^1^) than that with 50% Co (64.51 µmol h^−^^1^ mg^−^^1^), the latter shows significantly higher $F_{NH_{3}}$ (90.01% vs. 87.49%) and $S_{NH_{3}}$ (92.28% vs. 83.12%). Considering the overall catalytic performance, CuSn(OH)_6_/Co with 50% Co provides a better balance and is therefore selected as the representative catalyst for mechanistic investigations.

To further elucidate the catalytic behaviors, the optimized CuSn(OH)_6_/Co, pristine CuSn(OH)_6_, and Co nanoparticles were investigated in a 0.1 M Na_2_SO_4_ background electrolyte. As shown by the dashed lines in Figure 4d, the HER onset potential, defined as the potential required to reach a cathodic current density of 1.0 mA cm^−2^, is as low as −1.11 V for CuSn(OH)_6_, suggesting inferior HER activity [33, 34]. Remarkably, after introducing Co nanoparticles, the polarization curve shifts significantly toward positive potentials. The CuSn(OH)_6_/Co heterostructure exhibits an HER onset potential of −0.64 V, which is close to that of Co nanoparticles (−0.62 V) but substantially higher than CuSn(OH)_6_. Similarly, the HER Tafel slope of CuSn(OH)_6_/Co is determined to be 192.9 mV dec^−^^1^ (Figure S10), comparable to that of Co nanoparticles (173.1 mV dec^−^^1^) but significantly lower than that of CuSn(OH)_6_ (260.1 mV dec^−^^1^). These observations demonstrate that the HER activity of the heterostructure is dominated by the Co component [35, 36]. Given that metallic Co is well known for its capability to accelerate H_2_O dissociation, the incorporated Co nanoparticles can generate sufficient *H for NO_3_^−^RR [37, 38].

In the presence of 10 mM NaNO_3_, CuSn(OH)_6_ exhibits two reduction peaks at −0.33 and −0.88 V, which are assigned to the NO_3_^−^ → NO_2_^−^ and NO_2_^−^ → NH_3_ conversions [23], respectively. This result confirms that CuSn(OH)_6_ follows a stepwise nitrate reduction pathway involving the accumulation of NO_2_^−^ byproduct. The large peak potential difference ensures high activity and selectivity toward NO_2_^−^ production. However, the reduction peak associated with the NO_2_^−^ → NH_3_ reaction is located at a low potential of −0.88 V, suggesting that the second hydrogenation step is likely the rate‐limiting process for NH_3_ production on CuSn(OH)_6_. By contrast, Co nanoparticles display only a weak reduction peak centered at approximately −0.65 V, suggesting a direct NO_3_^−^‐to‐NH_3_ conversion pathway without significant accumulation of NO_2_^−^ byproduct. Impressively, the CuSn(OH)_6_/Co heterostructure also exhibits a single reduction peak located at approximately −0.50 V. The disappearance of the NO_2_^−^ reduction peak observed on pristine CuSn(OH)_6_ indicates that NO_2_^−^ generated on CuSn(OH)_6_ can be rapidly consumed at neighboring Co sites. Such behavior strongly supports the formation of a tandem catalytic mechanism within the heterostructure. Compared with pristine CuSn(OH)_6_, the NH_3_‐production peak of CuSn(OH)_6_/Co positively shifts by approximately 0.38 V. This substantial decrease in overpotential demonstrates that the incorporation of Co significantly facilitates the hydrogenation of nitrogen‐containing intermediates by supplying abundant *H through accelerated H_2_O dissociation, as evidenced by the enhanced HER activity (Figure 4d and Figure S10). Furthermore, the reduction peak of CuSn(OH)_6_/Co remains 0.15 V more positive than that of pure Co nanoparticles. This additional positive shift suggests that CuSn(OH)_6_ contributes to enhanced NO_3_^−^ adsorption and activation, thereby enhancing the overall activity. Consequently, the superior activity of CuSn(OH)_6_/Co originates from the synergistic coupling of NO_3_^−^ activation and *H supply functions at the heterointerface.

To further investigate the interfacial charge‐transfer behavior, electrochemical impedance spectroscopy (EIS) measurements were performed. As shown in Figure S11a, CuSn(OH)_6_/Co exhibits the lowest impedance magnitude among all samples, indicating enhanced electron transport and interfacial charge‐transfer kinetics [39]. The corresponding Bode phase plots (Figure S11b) reveal a lower maximum phase angle for CuSn(OH)_6_/Co, suggesting a faster electrochemical response and reduced kinetic limitations at the electrode/electrolyte interface [40]. The Nyquist plots in Figure 4e display characteristic semicircles for all electrodes, whose diameters correspond to the charge‐transfer resistance (Rct) [41]. Based on the equivalent circuit model shown in the inset of Figure 4e, the fitted R_ct_ value of CuSn(OH)_6_/Co is 51.4 Ω, substantially lower than those of CuSn(OH)_6_ (83.2 Ω) and Co nanoparticles (117.2 Ω). The significantly reduced R_ct_ verifies that strong electronic coupling between CuSn(OH)_6_ and Co facilitates electron migration across the heterointerface [29, 30]. Such efficient charge transport enables rapid electron delivery to adsorbed nitrogenous intermediates, thereby accelerating the overall NO_3_^−^RR kinetics. The electrochemically active surface area (ECSA) was subsequently evaluated by measuring cyclic voltammograms (CV) curves within a non‐Faradaic potential region at various scan rates (Figure S12). The linear relationship between double‐layer charging current density and scan rate is presented in Figure 4f, where the slope corresponds to the double‐layer capacitance (C_dl_). The CuSn(OH)_6_/Co exhibits a C_dl_ of 378.2 µF cm^−2^, which is higher than those of CuSn(OH)_6_ (345.1 µF cm^−2^) and Co (218.8 µF cm^−2^). The increased C_dl_ suggests that the introduction of Co nanoparticles not only constructs abundant heterointerfaces but also improves the accessibility of catalytic sites to the electrolyte [42]. Consequently, the CuSn(OH)_6_/Co heterostructure possesses a larger ECSA and a higher density of exposed active sites, both of which contribute to NH_3_ production [43]. Combined with the improved charge‐transfer kinetics and accelerated H_2_O dissociation capability, these characteristics collectively account for the outstanding NO_3_^−^RR activity and NH_3_ selectivity of the CuSn(OH)_6_/Co catalyst.

To further understand the catalytic behavior, bulk electrolysis was conducted over a potential range from −0.1 to −0.9 V with an interval of 0.1 V, and each electrolysis experiment was performed for 30 min. Comprehensive electrochemical metrics for NH_3_ and NO_2_^−^ products are provided in Tables S3–S8. As histogramatically shown in Figure 4g,i, the CuSn(OH)_6_ microspheres exhibit only a sluggish growth trend of $Y_{NH_{3}}$, $F_{NH_{3}}$, and $S_{NH_{3}}$ with increasing cathodic potential. Even at highly negative potentials, the NH_3_ production performance remains relatively limited, demonstrating that pristine CuSn(OH)_6_ possesses insufficient hydrogenation capability for efficient NH_3_ generation. In sharp contrast, the $Y_{{\mathrm{NO}}_{2}^{\;-}}$ and $F_{{\mathrm{NO}}_{2}^{\;-}}$ of CuSn(OH)_6_ increase dramatically as the applied potential turns more negative (Figure S13), achieving maximum values of 145.83 µmol h^−^^1^ mg_Cat_^−^^1^ and 77.94%, respectively, at −0.7 V. Moreover, CuSn(OH)_6_ maintains a high $S_{{\mathrm{NO}}_{2}^{\;-}}$ close to 100% over a broad potential window from −0.1 to −0.7 V. These observations unequivocally demonstrate that CuSn(OH)_6_ preferentially catalyzes the NO_3_^−^ reduction to *NO_2_, thereby accelerating the RDS in NO_3_^−^RR; whereas the subsequent hydrogenation of *NO_2_ intermediate is limited [23], in accordance with the polarization curve in Figure 4d. Therefore, CuSn(OH)_6_ can be regarded as a NO_3_^−^ activation catalyst to accelerate the RDS.

For Co nanoparticles, the $Y_{NH_{3}}$ tardily increases when lowering the electrode potential, whereas the $Y_{{\mathrm{NO}}_{2}^{\;-}}$ remains consistently low throughout the investigated potential range. In addition, both $F_{NH_{3}}$ and $S_{NH_{3}}$ of Co nanoparticles are substantially higher than the corresponding values for NO_2_^−^ formation. The negligible accumulation of NO_2_^−^ byproduct indicates that Co nanoparticles possess strong hydrogenation capability and can efficiently convert nitrogenous intermediates into NH_3_ [44]. Such behavior is consistent with the excellent H_2_O dissociation activity of metallic Co, as revealed by HER measurements [45]. Interestingly, the CuSn(OH)_6_/Co heterostructure exhibits a dramatically different catalytic behavior. The NH_3_ production indicators increase rapidly with negativing the electrode potential, reaching apex $Y_{NH_{3}}$ of 64.51 µmol h^−^^1^ mg_Cat_^−^^1^, $F_{NH_{3}}$ of 90.01%, and $S_{NH_{3}}$ of 92.28% at −0.7 V. These values are significantly higher than those of pristine CuSn(OH)_6_ ($Y_{NH_{3}}$ = 4.96 µmol h^−^^1^ mg_Cat_^−^^1^, $F_{NH_{3}}$ = 21.20%, $S_{NH_{3}}$ = 3.29%), highlighting the remarkable promotional effect of Co incorporation. More importantly, unlike pristine CuSn(OH)_6_, the $Y_{{\mathrm{NO}}_{2}^{\;-}}$, $F_{{\mathrm{NO}}_{2}^{\;-}}$, and $S_{{\mathrm{NO}}_{2}^{\;-}}$ of CuSn(OH)_6_/Co heterostructure maintains low over the entire potential range. The efficient suppression of NO_2_^−^ accumulation strongly suggests that the generated *NO_2_ intermediate are rapidly consumed at neighboring Co sites before diffusing into the bulk electrolyte. This behavior provides direct evidence for a tandem catalytic mechanism, in which CuSn(OH)_6_ preferentially converts NO_3_^−^ to *NO_2_, while Co nanoparticles dissociate H_2_O to produce *H and then accelerate the subsequent hydrogenation steps toward NH_3_ product. The key role of *H is supported by the tert‐butanol (TBA) quenching experiment, in which the TBA additive lowers catalytic current density and NH_3_ production (Figure S14) [46]. Moreover, by mixing CuSn(OH)_6_ and Co in a similar molar ratio, the resultant hybrid exhibits suppressed catalytic performance (Figure S15). This indicates that the heterointerfaces are crucial for the high catalytic activity of CuSn(OH)_6_/Co, possibly dominating the spillover of the *NO_2_ intermediate. At potentials more negative than −0.7 V, the $Y_{NH_{3}}$ of CuSn(OH)_6_/Co begins to decline. This decrease is attributed to the increasingly competitive HER, which consumes electrons and *H, thereby reducing the utilization efficiency of charge carriers for NO_3_^−^RR. Such behavior is consistent with the HER polarization curves shown in Figure 4d. At −0.7 V, the CuSn(OH)_6_/Co exhibits optimal NO_3_^−^RR to NH_3_ performance with a $Y_{NH_{3}}$ of 64.51 µmol h^−^^1^ mg_Cat_^−^^1^, a $F_{NH_{3}}$ of 90.01%, and a $S_{NH_{3}}$ of 92.28% in 10 mM NaNO_3_ and 0.1 M Na_2_SO_4_ electrolyte. Moreover, when raising the NaNO_3_ concentration to 0.1 M, CuSn(OH)_6_/Co also exhibits high catalytic performance with greatly improved catalytic current density and NH_3_ production performance ($Y_{NH_{3}}$ of 196.13 µmol h^−^^1^ mg_Cat_^−^^1^, $F_{NH_{3}}$ of 94.52%, and $S_{NH_{3}}$ of 92.49%), as shown in Figure S16. The catalytic performance achieved by CuSn(OH)_6_/Co outperforms or is comparable to the majority of the reported catalysts (Table S9).

To exclude potential contamination sources, control experiments were performed under nitrate‐free conditions and under open‐circuit potential (OCP). Negligible NH_3_ production was detected in both cases (Figure S17), confirming that the measured NH_3_ originates exclusively from electrochemical NO_3_^−^ reduction rather than environmental contamination. Moreover, to investigate the anti‐interference of CuSn(OH)_6_/Co toward common impurity ions, a series of control experiments was conducted to systematically investigate the effects of Cl^−^ and HCO_3_^−^. As shown in Figure S18a, the introduction of 10 mM Cl^−^, even at a high concentration of 0.1 M, has a negligible impact on the electrochemical behavior of CuSn(OH)_6_/Co, indicating its strong tolerance to Cl^−^. The $Y_{NH_{3}}$ increases with increasing Cl^−^ concentration due to the enhanced electrolyte conductivity (Figure S18b), while the $S_{NH_{3}}$ remains at a high level upon the introduction of Cl^−^. In contrast, the introduction of HCO_3_^−^ substantially suppresses NO_3_^−^RR current density, accompanied by a pronounced decrease in $Y_{NH_{3}}$ (Figure S18c,d). The decreased catalytic performance is largely due to the competitive adsorption of HCO_3_^−^, thus impeding NO_3_^−^ adsorption [47]. Additionally, CuSn(OH)_6_/Co maintains high catalytic performance in simulated nitrate wastewater with 10 mM NaNO_3_ in natural lake water (Figure S19), achieving a $Y_{NH_{3}}$ of 42.61 µmol h^−^^1^ mg_Cat_^−^^1^, a $F_{NH_{3}}$ of 89.68%, and a $S_{NH_{3}}$ of 92.14%. These results further demonstrate the excellent anti‐interference capability of CuSn(OH)_6_/Co toward common impurity ions in practical nitrate‐containing wastewater.

**FIGURE 5:**
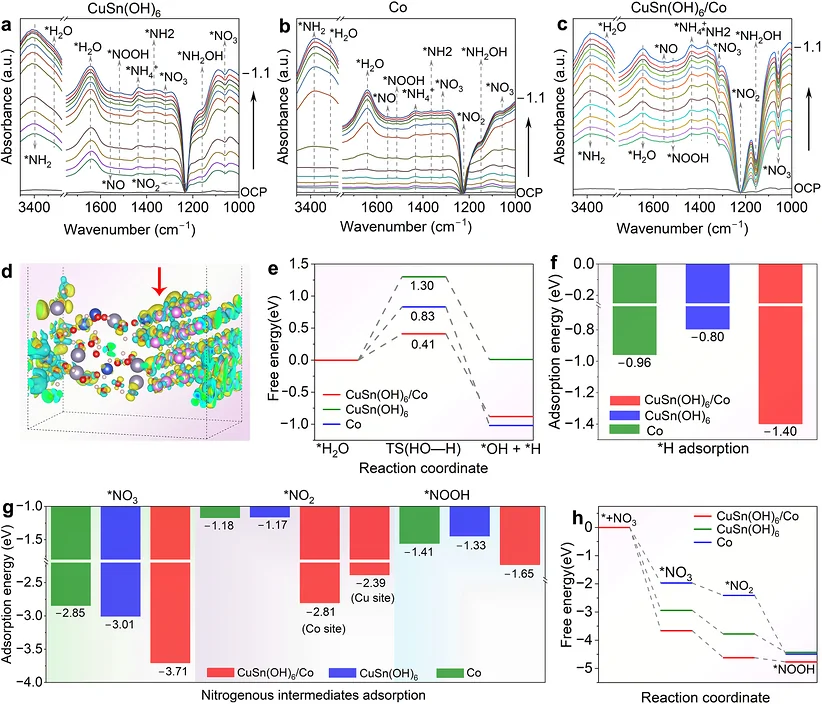
In situ ATR‐FTIR spectra of (a) CuSn(OH)_6_, (b) Co, and (c) CuSn(OH)_6_/Co. (d) Charge density difference (CDD) of the CuSn(OH)_6_/Co heterostructure. Cyan and yellow isosurfaces represent charge depletion and charge accumulation, respectively. (e) Free‐energy profiles for H_2_O dissociation on CuSn(OH)_6_, Co, and CuSn(OH)_6_/Co. (f) Calculated *H adsorption energies for the three catalysts. (g) Adsorption energies of key nitrogen‐containing intermediates (*NO_3_, *NO_2_, *NOOH) over CuSn(OH)_6_, Co, and CuSn(OH)_6_/Co. (h) Gibbs free‐energy diagram of the three catalysts for the reaction path: * + NO_3_^−^ → *NO_3_ → *NO_2_ → *NOOH.

To elucidate the catalytic mechanism, in situ attenuated total reflection Fourier‐transform infrared (ATR‐FTIR) spectroscopy analyses were performed. As shown in Figure 5a, CuSn(OH)_6_ exhibits obvious downward peaks at ∼1060 and ∼1315 cm^−^^1^, corresponding to the symmetric and asymmetric stretching vibrations of N–O bonds in *NO_3_ [48, 49, 50], respectively. The decrease in equilibrium surface concentration of *NO_3_ is attributed to CuSn(OH)_6_ effectively adsorbing and activating NO_3_^−^ and then efficiently catalysing to *NO_2_. The pronounced downward peak at ∼1225 cm^−^^1^ is assigned to the *NO_2_ intermediate, indicating its continuous formation and subsequent consumption on the catalyst surface [51]. The prominent peak at 1645 cm^−^^1^ and the shoulder peak at 3258 cm^−^^1^ are associated with H–O–H bending and O–H stretching vibrations of adsorbed *H_2_O [52, 53], respectively. In addition, characteristic signals associated with key nitrogen‐containing intermediates, including *NH_2_OH (∼1155 cm^−^^1^), *NO (∼1552 cm^−^^1^), *NOOH (∼1516 cm^−^^1^),*NH_2_ (∼1365 and ∼3375 cm^−^^1^), and *NH_4_^+^ (∼1433 cm^−^^1^), are also observed on CuSn(OH)_6_. In contrast, the peaks associated with *NO_3_ on Co nanoparticles are very weak, indicating their poor capability for NO_3_^−^ adsorption and activation (Figure 5b). Compared with CuSn(OH)_6_, Co nanoparticles exhibit a pronounced increase in the intensity of the characteristic *H_2_O peaks as the electrode potential becomes more negative. This result indicates that Co nanoparticles possess a stronger ability to adsorb and activate H_2_O, thereby promoting water dissociation and generating more *H species for the hydrogenation of nitrogen‐containing intermediates. In contrast, CuSn(OH)_6_/Co shows more pronounced *NO_3_‐related signals compared with CuSn(OH)_6_ (Figure 5c). More importantly, compared with those of CuSn(OH)_6_ and Co nanoparticles, the peak at ∼1155 cm^−^^1^ for CuSn(OH)_6_/Co decreases more rapidly with increasing cathodic potential, indicating accelerated consumption of the *NH_2_OH intermediate owing to the fast electrode kinetics [54]. Furthermore, CuSn(OH)_6_/Co exhibits a higher intensity of the *NH_4_^+^ signal at ∼1433 cm^−^^1^ than either CuSn(OH)_6_ or Co nanoparticles, further confirming that the heterostructure promotes the overall NO_3_^−^RR kinetics and facilitates the formation of NH_3_.

To gain deep insight into the catalytic mechanism of CuSn(OH)_6_/Co, density functional theory (DFT) calculations were performed. The structure models for CuSn(OH)_6_, Co, and CuSn(OH)_6_/Co are shown in Figure S20. CuSn(OH)_6_/Co exhibits an increased density of states (DOS) near the Fermi level compared with the individual components (Figure S21), indicating enhanced electronic interactions and electronic‐state modulation at the heterointerface. The Cu and Co d‐band centers in CuSn(OH)_6_/Co are located at −2.545 and −2.176 eV, respectively, both slightly upshifted relative to CuSn(OH)_6_ (−2.558 eV) and Co (−2.269 eV). This upshift indicates modified electronic structures of the interfacial active sites, which can facilitate their interactions with reaction intermediates [55]. The charge density difference (CDD) analysis reveals pronounced charge accumulation at the CuSn(OH)_6_ side near the heterointerface (shown by the red arrow in Figure 5d), confirming the electron transfer from Co to CuSn(OH)_6_ and strong electronic coupling of the heterostructure.

Since *H generated through H_2_O dissociation is essential for the reduction of NO_3_^−^ to NH_3_, the H_2_O dissociation behavior was further investigated [56]. As the Sn sites are not the active centers, H_2_O adsorption and dissociation on the Cu sites of CuSn(OH)_6_ were evaluated (Figure S22) [23]. As shown in Figure 5e, the H_2_O dissociation energy decreases from 1.30 eV on CuSn(OH)_6_ to 0.83 eV on Co and further to 0.41 eV on the Co site of CuSn(OH)_6_/Co, demonstrating that the heterointerface markedly promotes H_2_O activation [56]. Meanwhile, the corresponding *H adsorption energy becomes more favorable on the heterostructure (−1.41 eV) than on Co (−0.96 eV) and CuSn(OH)_6_ (−0.80 eV) (Figure 5f and Figure S23). These results indicate that the Co component, particularly after coupling with CuSn(OH)_6_, is favorable for H_2_O dissociation and *H adsorption, providing a favorable hydrogenation environment for NO_3_^−^ reduction. The adsorption behaviors of key nitrogen‐containing intermediates further reveal the distinct catalytic roles of the two components (Figures S24–S26). As shown in Figure 5g, the Cu site of CuSn(OH)_6_/Co shows the strongest *NO_3_ adsorption (−3.71 eV), compared with CuSn(OH)_6_ (−3.01 eV) and Co (−2.85 eV), indicating favorable NO_3_^−^ adsorption at the heterointerface [57]. Notably, *NO_2_ binds much more strongly to the Co site of CuSn(OH)_6_/Co (−2.81 eV) than to the Cu site of CuSn(OH)_6_/Co (−2.39 eV), CuSn(OH)_6_ (−1.17 eV), and Co (−1.18 eV), suggesting that *NO_2_ formed on CuSn(OH)_6_ can be preferentially transferred toward the Co component via the heterointerface. Consistently, the Co site of CuSn(OH)_6_/Co exhibits a more favorable *NOOH adsorption (−1.65 eV) than CuSn(OH)_6_ (−1.33 eV), and Co (−1.41 eV), ensuring subsequent hydrogenation to NH_3_ on the heterostructure. The calculated reaction energetics further support this interfacial tandem mechanism. As shown in Figure 5h, CuSn(OH)_6_ exhibits more favorable energetics for NO_3_^−^ → *NO_3_ (−2.94 eV) and *NO_3_ → *NO_2_ (−0.84 eV) than Co, whereas Co shows a lower free‐energy change for *NO_2_ → *NOOH (−2.08 eV) than CuSn(OH)_6_ (−0.65 eV). These results support a tandem catalytic pathway on CuSn(OH)_6_/Co: 1) CuSn(OH)_6_ preferentially adsorbs and activates NO_3_^−^ and promotes its conversion to *NO_2_; 2) *NO_2_ migrates from CuSn(OH)_6_ to Co across the heterointerfaces; 3) Co facilitates H_2_O dissociation to generate *H and promotes *NO_2_ hydrogenation to *NOOH. Once *NOOH is formed, further hydrogenation to NH_3_ is effortless. Therefore, other nitrogen‐containing intermediates are not theoretically investigated here [58].

**FIGURE 6:**
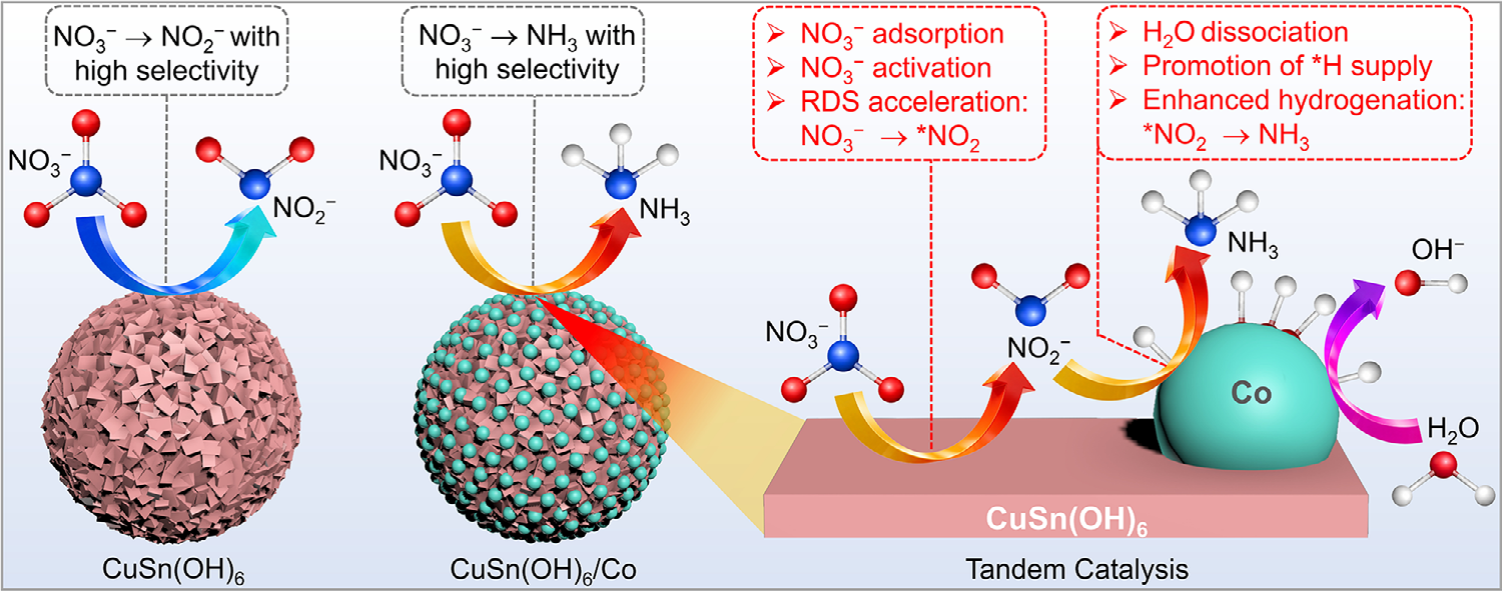
NO_3_^−^RR mechanisms over CuSn(OH)_6_ and CuSn(OH)_6_/Co heterostructure.

Collectively, the experimental and theoretical results unequivocally demonstrate that CuSn(OH)_6_ and Co play distinct yet highly complementary catalytic roles within the heterostructure. Pristine CuSn(OH)_6_ microspheres convert NO_3_^−^ to *NO_2_ with high intrinsic activity and selectivity, originating from the unique chemical composition and crystal structure. However, the further conversion of NO_2_^−^ to NH_3_ over CuSn(OH)_6_ is impeded by the weak hydrogenation activity. In contrast, Co nanoparticles exhibit superior H_2_O activation and can efficiently generate abundant *H on their surfaces, thereby facilitating the rapid hydrogenation of the *NO_2_ intermediate to NH_3_. Nevertheless, pure Co nanoparticles exhibit relatively weak NO_3_^−^ adsorption and activation capabilities, rendering the initial NO_3_^−^ → NO_2_^−^ conversion inefficient and consequently restricting overall NO_3_^−^RR performance. By integrating these complementary functionalities, the CuSn(OH)_6_/Co heterostructure establishes an efficient interfacial tandem‐catalysis pathway for selective NH_3_ synthesis. As illustrated schematically in Figure 6, CuSn(OH)_6_ adsorbs and activates NO_3_^−^ efficiently to promote the *NO_3_ → *NO_2_ reaction, thus accelerating the RDS of NO_3_^−^RR dramatically. Next, the *NO_2_ spills over from CuSn(OH)_6_ to Co. Subsequently, ensured by sufficient *H supply from Co nanoparticles due to facile H_2_O dissociation, the *NO_2_ intermediates are exclusively reduced to NH_3_ product on the nearby Co sites. Such spatial separation of catalytic functions not only optimizes each elementary reaction step but also enables efficient intermediate channeling between neighboring active sites. As a result, NO_2_^−^ accumulation is effectively suppressed, while the overall reaction pathway is directed toward complete NO_3_^−^ → NH_3_ conversion. Therefore, the superior performance of the CuSn(OH)_6_/Co catalyst originates from the synergistic coupling of accelerated NO_3_^−^ activation and enhanced NO_2_^−^ hydrogenation, highlighting the effectiveness of interfacial tandem catalysis in regulating reaction kinetics and product selectivity during NO_3_^−^RR.

**FIGURE 7:**
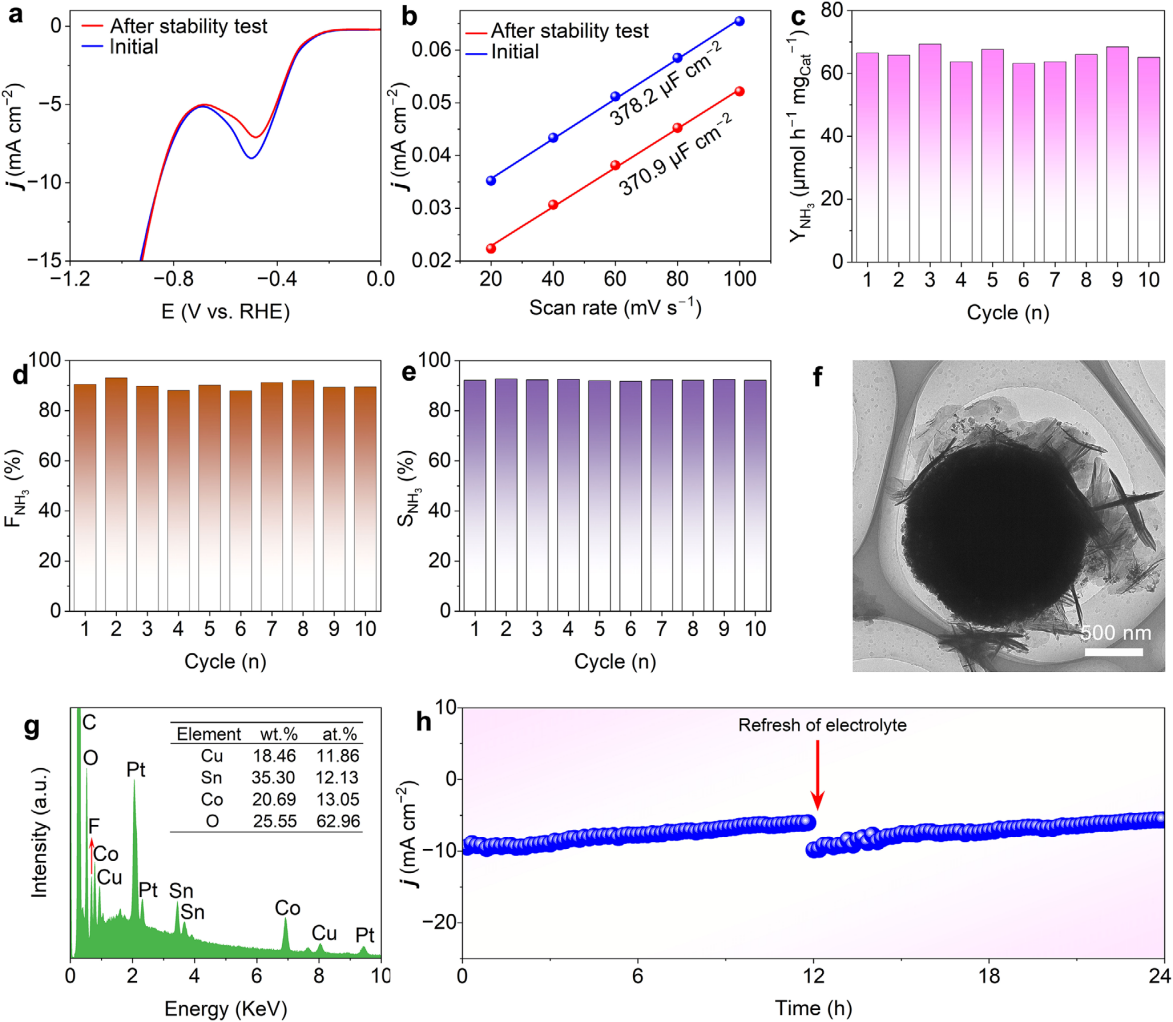
(a) Polarization curves and (b) C_dl_ of CuSn(OH)_6_/Co before and after the stability test. (c) $Y_{NH_{3}},$ (d) $F_{NH_{3}},$ and (e) $S_{NH_{3}}$ of CuSn(OH)_6_/Co during the 10 cycles. (e) TEM image of CuSn(OH)_6_/Co acquired after stability test. (g) EDS spectrum and corresponding element content of Cu(OH)_2_/Co after the stability measurement. (h) Chronoamperometry curve of CuSn(OH)_6_/Co recorded at −0.7 V.

To evaluate the durability of the catalyst, the CuSn(OH)_6_/Co electrode was repeatedly tested at −0.7 V for 30 min per cycle. After 10 consecutive cycles, the polarization curve exhibits negligible attenuation in catalytic current density compared with the initial cycle (Figure 7a). Notably, the characteristic reduction peak associated with rapid NO_3_^−^‐to‐NH_3_ conversion remains essentially unchanged, indicating that the intrinsic catalytic functionality of the heterostructure is well preserved. Meanwhile, the C_dl_ remains nearly identical to that of the fresh electrode (Figure 7b and Figure S27). The negligible variation in C_dl_ suggests that the ECSA remains stable during repeated operation, without significant deactivation of active sites occurring. Further quantitative analysis of the catholyte indicates that the $Y_{NH_{3}},$ $F_{NH_{3}},$ and $S_{NH_{3}}$ are retained at their initial level throughout the cycling tests (Figure 6c–e), while the $Y_{{\mathrm{NO}}_{2}^{\;-}}$, $F_{{\mathrm{NO}}_{2}^{\;-}}$, and $S_{{\mathrm{NO}}_{2}^{\;-}}$ remain consistently low (Figure S28). These electrochemical investigations suggest that the NO_3_^−^ reduction to NH_3_ activity of CuSn(OH)_6_/Co is preserved during cycling, demonstrating the effectiveness of the tandem catalytic pathway. Post‐reaction TEM measurements suggest the retention of microspherical morphology and heterostructure (Figure 7f). SEM image and elemental mapping results further confirm the retention of its original morphology and homogeneous elemental distribution (Figure S29). Moreover, the elemental compositions determined by the EDS spectrum remain essentially unchanged compared with those of the fresh catalyst (Figure 7g), documenting the high structural and compositional stability of the CuSn(OH)_6_/Co heterostructure. Further XPS analyses reveal minimal changes in surface composition after cycling (Figure S30). Additionally, the chronoamperometric measurement at −0.7 V suggests that the CuSn(OH)_6_/Co runs smoothly for 24 h (Figure 7h), without notable decay in catalytic activity (Figure S31). As a result, the CuSn(OH)_6_/Co catalyst represents a highly efficient and durable catalyst for practical NH_3_ production via NO_3_^−^RR.

## 3 Conclusion

In summary, we have successfully constructed a CuSn(OH)_6_/Co heterostructure as an efficient electrocatalyst for selective NO_3_^−^ reduction to NH_3_ through an interfacial tandem‐catalysis strategy. In the heterostructure, CuSn(OH)_6_ adsorbs and activates NO_3_^−^ and then promotes its conversion to *NO_2_. Afterward, *NO_2_ migrates to the Co component. Simultaneously, Co nanoparticles facilitate H_2_O dissociation and provide abundant *H for the subsequent hydrogenation of the *NO_2_ intermediate to NH_3_, forming a tandem catalytic pathway. Benefiting from the synergistic interaction across the heterointerface, the directional transfer and rapid consumption of the *NO_2_ intermediate are significantly enhanced, effectively suppressing intermediate accumulation and improving NH_3_ selectivity. As a result, the CuSn(OH)_6_/Co catalyst achieves an NH_3_ yield rate of 64.51 µmol h^−^^1^ mg_Cat_^−^^1^ with an NH_3_ selectivity of 92.28% at −0.7 V in 0.1 M in 0.1 M NaSO_4_ electrolyte with 10 mM NaNO_3_. This work establishes interfacial tandem catalysis as an effective strategy to decouple and optimize the individual elementary steps of NO_3_^−^RR, offering new opportunities for engineering reaction pathways through spatially organized active sites. The insights gained herein may inspire the rational design of high‐performance electrocatalysts for green NH_3_ production from NO_3_^−^RR.

## Author Contributions

**Yuan‐Yuan Hei**: writing – original draft, resources, conceptualization. **Qing Chen**: methodology, investigation, writing – original draft. **Jie Gu**: validation, data curation. **Yifan Leng**: data curation, validation. **Yaping Huang**: methodology. **Sunhui Chen**: methodology. **Hong Sun**: resources, data curation. **Hongfang Du**: writing – review and editing, supervision, project administration, resources, funding acquisition.

## Conflicts of Interest

The authors declare no conflicts of interest.

## Supporting information




**Supporting File**: advs77640‐sup‐0001‐SuppMat.docx.

## Data Availability

The data that support the findings of this study are available from the corresponding author upon reasonable request.
