# Supplementary material for: Interfacial Tandem Catalysis Over a CuSn(OH)6/Co Heterostructure Enables Efficient Electroreduction of Nitrate to Ammonia
Source: Adv Sci (Weinh). 2026 Sep 8:e77640. Online ahead of print. doi: 10.1002/advs.77640 (PMC13554498; doi:10.1002/advs.77640)
Supplement: Supplementary file 1 [file ADVS-9999-e77640-s001.docx]

Supporting Information

Interfacial Tandem Catalysis over a CuSn(OH)₆/Co Heterostructure Enables Efficient Electroreduction of Nitrate to Ammonia

Yuan-Yuan Hei, Qing Chen, Jie Gu, Yifan Leng, Yaping Huang, Sunhui Chen, Hong Sun* and Hongfang Du*

Y. Hei, Y. Huang, S. Chen, H. Sun

Department of Pharmacy, Fuzhou University Affiliated Provincial Hospital, Fuzhou University, Fuzhou, 350001, China

E-mail: sunhong7777@fjmu.edu.cn

Q. Chen, J. Gu, Y. Leng, H. Du

Strait Laboratory of Flexible Electronics (SLoFE), Strait Institute of Flexible Electronics (SIFE, Future Technologies), Fujian Normal University, Fuzhou 350117, China

E-mail: ifehfdu@fjnu.edu.cn

**1 Experimental Section**

1.1. Material Synthesis

CuSn(OH)₆ microspheres were synthesized according to our previous work, as detailed elsewhere (ACS Sustainable Chem. Eng. 2025,1 3, 4046−4054). Next, CoSO₄·7H₂O powder was dissolved in 30 mL of deionized (DI) water. The CuSn(OH)₆ microspheres were then dispersed in CoSO₄ solution with the help of an ultrasonication bath. The dosages of both CoSO₄·7H₂O and CuSn(OH)₆ microspheres were controlled to no more than 1.0 mmol. Subsequently, 20 mL of 0.5 M NaBH₄ solution was added dropwise to the above suspension. After stirring for 2 h, CuSn(OH)₆/Co was thoroughly washed with DI water until the washing solution reached neutral pH and no NH₃ was detected. The resulting CuSn(OH)₆/Co was then dried in a vacuum oven at 70 °C for 12 h. The CuSn(OH)₆/Co with various molar ratios was synthesized by tuning the feed ratio of CoSO₄·7H₂O and CuSn(OH)₆ microspheres. The Co nanoparticles were synthesized in identical experimental procedures, except for the absence of CuSn(OH)₆ microspheres.

1.2. Physical characterization

Scanning electron microscopy (SEM) and energy-dispersive X-ray spectroscopy (EDS) characterizations were conducted using a field-emission scanning electron microscope (FEI Verios G4). Transmission electron microscopy (TEM) analysis was carried out on a TALOS F200X instrument. Powder X-ray diffraction (XRD) data were collected on a D8 ADVANCE diffractometer with Cu Kα radiation. X-ray photoelectron spectroscopy (XPS) measurements were performed using a Shimadzu Kratos AXIS Ultra DLD system, with all binding energies calibrated against the C 1s peak at 284.8 eV. UV–vis absorption spectra were acquired on a Hitachi UH5700 UV–Vis–NIR spectrophotometer. Gas chromatography (GC) measurements were performed using a FULI GC9790plus gas chromatograph equipped with thermal conductivity (TCD) and flame ionization (FID) detectors, with argon as the carrier gas. In situ attenuated total reflection Fourier transform infrared spectroscopy (ATR-FTIR) analyses were performed with a Nicolet iS50 FTIR spectrometer equipped with attenuated total reflection (ATR) accessories.

1.3. Electrochemical measurements

A total of 6.25 mg of catalyst was ultrasonically dispersed for 30 minutes in a mixed solvent containing 480 μL of ethanol and 20 μL of 5 wt% Nafion solution to form a homogeneous ink. Subsequently, 10 μL of the suspension was drop-cast onto a carbon cloth electrode (geometric area: 0.5 × 0.5 cm²) pre-sealed with molten wax, resulting in a catalyst loading of approximately 0.5 mg cm⁻². Electrochemical measurements were carried out on a CHI760D electrochemical workstation using an H-type cell separated by a proton exchange membrane. A saturated calomel electrode (SCE) and a platinum foil served as the reference and counter electrodes, respectively. The electrolyte consisted of an aqueous solution containing 0.1 M Na₂SO₄ (99%, Energy Chemical) and 10 mM NaNO₃ (99%, Aladdin). All potentials were measured versus SCE and subsequently converted to the reversible hydrogen electrode (RHE) scale according to: E_RHE_ = E_SCE_ + 0.242 + 0.0591 × pH. Electrochemical impedance spectroscopy (EIS) measurements were conducted at −0.5 V over a frequency range from 0.01 Hz to 1000 Hz with an AC amplitude of 5 mV. The polarization curves were recorded at a scan rate of 5 mV s⁻¹, and chronoamperometric electrolysis was performed at selected potentials for 30 minutes.

1.4. Product analysis

*Quantification of NH_3_:* The concentration of NH_3_ product was quantified using the indophenol blue colorimetric method. Following electrolysis, 1.0 mL of the catholyte was diluted with 4.0 mL of deionized (DI) water. Subsequently, 2.0 mL of an alkaline solution containing 1.0 M NaOH (97%, Energy Chemical), 5 wt.% salicylic acid (99%, Energy Chemical), and 5 wt.% sodium citrate (99%, Energy Chemical) was introduced into the mixture. Then, 1.0 mL of 0.05 M NaClO solution (10% active chlorine, Energy Chemical) and 0.2 mL of 1 wt.% sodium nitroferricyanide (99%, Energy Chemical) were sequentially added. The resulting solution was allowed to react in the dark for 1 h to complete color development. UV–Vis absorption spectra were then collected, and the absorbance at 654 nm was used to determine the NH_3_ concentration. Calibration was performed using NH_4_Cl standard solutions with known concentrations.

*Quantification of* *NO_2_^−^*: The concentration of NO_2_^−^ was measured by the Griess colorimetric method. The chromogenic reagent was prepared by dissolving 4.0 g p-aminobenzenesulfonamide (99%, Energy Chemical and 0.2 g N-(1-naphthyl)ethylenediamine dihydrochloride (97%, Energy Chemical) in a mixture containing 10 mL phosphoric acid (85 wt.%) and 50 mL DI water. After electrolysis, 1.0 mL of catholyte was diluted to 50 mL with DI water. Subsequently, 0.1 mL of color reagent was added to 5.0 mL of the diluted sample. After incubation for 20 minutes at room temperature, the absorbance spectrum was recorded by UV–Vis spectroscopy. The absorbance at 540 nm was employed for quantitative determination, and a standard calibration curve was established using KNO_2_ solutions with known concentrations.

*Quantification of N_2_H_4_:* Hydrazine was analyzed using the p-(dimethylamino)benzaldehyde colorimetric method. The color-developing reagent was prepared by dissolving 2.0 g p-(dimethylamino)benzaldehyde (99%, Energy Chemical) in a mixed solution containing 100 mL of ethanol and 12 mL of 1 M HCl. For analysis, 0.2 mL of the reagent was added to 5.0 mL of catholyte and allowed to react for 15 minutes. The absorbance at 457 nm was subsequently measured by UV–Vis spectroscopy. Standard N_2_H_4_ solutions with varying concentrations were employed to construct the calibration curve.

1.5. Calculation of Product Yield (Y), Selectivity (S), and Faradaic Efficiency (F)

The yields of NH_3_ and NO_2_^−^ generated during bulk electrolysis were calculated according to the following equations:

Y(μg h^−1^ mg_Cat_^−1^) = (*C* × *V*) / (*m* × *t*)

Y (μmol h^−1^ mg_Cat_^−1^) = Y (μg h^−1^ mg_Cat_^−1^)/*M*

where *C* is the experimentally determined concentration of NH_3_ or NO_2_^−^ (mg·mL⁻¹); *V* is the electrolyte volume (mL); *m* is the is catalyst loading (mg); *t* is the electrolysis time (h); *M* is the molecular weight of the target product (17 g·mol^−1^ for NH_3_ and 46 g·mol^−1^ for NO_2_^−^).

Because NH₃ and NO₂⁻ were the only nitrogen-containing products detected during nitrate reduction, the product selectivity was determined from their corresponding molar yields using the following equations:

$S_{{NH}_{3}}$= $Y_{{NH}_{3}}$/($Y_{{NO}_{2^{-}}}$ + $Y_{{NH}_{3}}$)

$S_{{NO}_{2^{-}}}$= $Y_{{NO}_{2^{-}}}$/($Y_{{NO}_{2^{-}}}$ + $Y_{{NH}_{3}}$)

where $S_{{NH}_{3}}$ and $S_{{NO}_{2^{-}}}$ represents the selectivities of NH_3_ and NO_2_^−^, respectively, while $Y_{{NH}_{3}}$ and $Y_{{NO}_{2^{-}}}$ are the corresponding molar yields of NH_3_ and NO_2_^−^.

The Faradaic efficiency (F) for NH₃ and NO₂⁻ products was calculated based on the following equations:

$F_{{NH}_{3}}$ = [($n_{{NH}_{3}}$ × 8*F*)/*Q*] × 100%

$F_{{NO}_{2^{-}}}$ = [($n_{{NO}_{2^{-}}}$ × 2*F*)/*Q*] × 100%

where $n_{{NH}_{3}}$ and $n_{{NO}_{2^{-}}}$ are the amounts of NH₃ and NO₂⁻ produced (mol), respectively, *F* is the Faraday constant (96485 C mol⁻¹), and *Q* is the total charge passed during electrolysis (C). The values 8 and 2 correspond to the number of electrons required for the reduction of nitrate to NH₃ and NO₂⁻, respectively.

1.6. Computational method

All calculations were carried out for the material in the framework of Density Functional Theory (DFT) using the Vienna Ab initio Simulation Package (VASP) (Phys. Rev. B, 1996, 54, 11169-11186; Phys. Rev. B, 1999, 59, 1758-1775). The CuSn(OH)₆, Co, and CuSn(OH)₆/Co structure models were constructed from standard crystal information files (ICSD#194 for CuSn(OH)₆ and ICSD#53805for Co). Hydrogen atoms for the CuSn(OH)₆‑related models were manually added. The exchange–correlation interactions were described using the generalized gradient approximation (GGA) with the Perdew–Burke–Ernzerhof (PBE) functional (Phys. Rev. Lett. 1996, 77, 3865–3868). The projector augmented-wave (PAW) method was employed to describe the electron–ion interactions (Phys. Rev. B 1994, 50, 17953–17979). A plane-wave energy cutoff of 500 eV was used for all calculations. A Monkhorst–Pack k-point mesh of 4 × 4 × 1 was adopted for the slab and heterostructure models. Spin-polarized calculations were performed for all Co-containing systems. To account for the localized d electrons of the transition-metal atoms, the DFT+U approach within the Dudarev formalism was employed, with effective U values (Ueff = U − J) of 2.5 and 2.0 eV applied to the Co 3d and Cu 3d states, respectively. The electronic self-consistent field (SCF) convergence criterion was set to 10⁻⁶ eV, and the structural optimization was considered converged when the residual forces on all unconstrained atoms were below 0.03 eV Å⁻¹. The lattice parameters and atomic positions of the bulk structures were fully relaxed, whereas the lattice parameters of the slab and heterostructure models were fixed and the atomic positions were relaxed accordingly.

2 Supporting figures

Figure S1. XRD patterns of CuSn(OH)₆ microspheres and Co nanoparticles.

Figure S2. (a, b) SEM, (c, d) TEM, (e) HRTEM images of CuSn(OH)₆ microspheres.

Figure S3. EDS spectrum and corresponding elemental content of Cu(OH)_2_/Co sample. The Pt signals originate from Pt sputtering prior to SEM measurement, which was performed to eliminate the charging effect and improve imaging clarity.

Figure S4. (a, b) SEM, (c) TEM, (d) HRTEM images of Co nanoparticles.

**Figure S5** (a) UV-Vis spectrum and (b) absorbance at 654 nm of the standard NH_3_ solution with various concentrations.

**Figure S6.** (a) UV-Vis spectra and (b) absorbance at 540 nm for the standard NaNO_2_ solution with various concentrations.

**Figure S7.** (a) UV-Vis spectra and (b) absorbance at 457 nm of standard N_2_H_4_ solution with various concentrations.

**Figure S8.** (a) UV-Vis spectra of CuSn(OH)_6_/Co acquired after bulk electrolysis at −0.7 V for 30 minutes.

**Figure S9.** Gas product analysis of CuSn(OH)_6_/Co after bulk electrolysis at −0.7 V for 30 minutes via gas chromatography.

**Figure S10.** HER Tafel plots of CuSn(OH)₆/Co, CuSn(OH)₆, and Co acquired in 0.1 M Na_2_SO_4_ electrolyte.

**Figure S11**. **(a)** Bode impedance magnitude and **(b)** Phase angle as a function of frequency for CuSn(OH)₆/Co , CuSn(OH)₆, and Co.

**Figure S12.** CV curves of (a) CuSn(OH)_6_/Co, (b) Co, and (c) CuSn(OH)_6_ acquired at scan rates of 20, 40, 60, 80, and 100 mV s^−1^. The current density ***j*** at the central position was chosen to calculate the double-layer capacitance, ***j*** = 0.5 (***j***_a_ - ***j***_c_).

**Figure S13** (a) $\text{Y}_{\text{NO}_{\text{2}}^{\text{−}}}$, (b) $\text{F}_{\text{NO}_{\text{2}}^{\text{−}}}$, and $\text{S}_{\text{NO}_{\text{2}}^{\text{−}}}$ of CuSn(OH)_6_/Co, CuSn(OH)_6_, and Co.

**Figure S14** (a) Polarization curves of CuSn(OH)_6_/Co in the presence of various amounts of tert-butanol (TBA) additive. (b) NH_3_ product analysis of CuSn(OH)_6_/Co in the presence of in the presence of 10 mM TBA.

**Figure S15** (a) Comparison of polarization curves of CuSn(OH)_6_ and Co hybrid (in molar ratio of 1:1) with CuSn(OH)_6_/Co. (b) NH_3_ product analysis of CuSn(OH)_6_ and Co hybrid.

**Figure S16** (a) Polarization curves of CuSn(OH)_6_/Co measured in 0.1 M NaNO_3_ and 0.1 M Na_2_SO_4_ electrolyte. (b) NH_3_ product analysis of CuSn(OH)_6_/Co after bulk electrolysis at −0.7 V for 30 minutes in 0.1 M NaNO_3_ and 0.1 M Na_2_SO_4_ electrolyte.

**Figure S17.** $\text{Y}_{\text{NH}_{\text{3}}}$ comparison of CuSn(OH)_6_/Co catalyst measured with or without 10 mM NaNO_3_ at −0.7 V for 30 minutes, and at OCP condition for 30 minutes.

**Figure S18.** CV curves of CuSn(OH)_6_/Co after the stability test.

**Figure S19.** (a, b) Map and geographical location of Xingyu Lake at the Qishan Campus of Fujian Normal University. (c) Photograph of Xingyu Lake. (d) LSV curve and (e) NH₃ production of CuSn(OH)₆/Co in simulated nitrate wastewater at −0.7 V for 30 min. The nitrate wastewater was simulated by adding NaNO₃ to natural water collected from Xingyu Lake to a final nitrate concentration of 10 mM.

**Figure S20.** Structure models for (a) CuSn(OH)_6_, (b) Co, and (c) CuSn(OH)₆/Co.

**Figure S21.** Density of states (DOS) of (a) CuSn(OH)_6_, (b) Co, and (c) CuSn(OH)₆/Co.

**Figure S22.** H_2_O dissociation on (a, b) CuSn(OH)_6_, (c, d) Co, and (e, f) CuSn(OH)₆/Co.

**Figure S23.** Optimized *H adsorption configuration of (a) CuSn(OH)_6_, (b) Co, and (c) CuSn(OH)₆/Co.

**Figure S24.** Optimized configurations for (a) *NO_3_, (b) *NO_2_, and (c) *NOOH adsorption on CuSn(OH)_6_.

**Figure S25.** Optimized configurations for (a) *NO_3_, (b) *NO_2_, and (c) *NOOH adsorption on Co.

**Figure S26.** Optimized configurations for (a) *NO_3_ on the Cu site, (b) *NO_2_ on the Cu site, (c) *NO_2_ on the Co site, and (d) *NOOH adsorption on the Cu site of CuSn(OH)_6_/Co.

**Figure S27.** CV curves of CuSn(OH)_6_/Co after the stability test.

**Figure S28.** (a) $\text{Y}_{\text{NO}_{\text{2}}^{\text{−}}}$, (b) $\text{F}_{\text{NO}_{\text{2}}^{\text{−}}}$, and $\text{S}_{\text{NO}_{\text{2}}^{\text{−}}}$ of CuSn(OH)_6_/Co after the stability test.

**Figure S29.** (a) SEM and (b-f) corresponding EDS mapping images of CuSn(OH)_6_/Co after the stability measurement.

**Figure S30.** (a) Survey, (b) Sn 3d, (c) Cu 2p, and (d) Co 2p XPS spectra of CuSn(OH)_6_/Co after the stability measurement.

**Figure S31.** Polarization curves of CuSn(OH)_6_/Co recorded at various periods during chronoamperometry measurements.

**3 Supporting tables**

**Table S1.** NH_3_ product analysis of Cu(OH)_6_/Co catalyst with various Co content at −0.7 V.

| Co content  (%) | $\text{Y}_{\text{NH}_{\text{3}}}$  (μmol h^−1^ mg^−1^) | $\text{F}_{\text{NH}_{\text{3}}}$  (%) | $\text{S}_{\text{NH}_{\text{3}}}$  (%) |
| --- | --- | --- | --- |
| 0 | 4.96±0.14 | 21.20±1.53 | 3.29±0.10 |
| 1 | 38.90±0.93 | 71.80±2.43 | 60.09±1.24 |
| 2 | 55.61±1.26 | 86.50±2.27 | 76.06±1.09 |
| 10 | 69.64±1.70 | 87.49±2.55 | 83.12±0.60 |
| 20 | 66.19±1.64 | 89.15±2.04 | 89.36±0.49 |
| 50 | 64.51±1.57 | 90.01±2.57 | 92.28±0.45 |
| 70 | 57.38±1.92 | 90.53±2.29 | 92.73±0.58 |
| 90 | 46.93±1.47 | 89.26±2.49 | 92.93±0.41 |
| 100 | 4.60±0.11 | 90.04±2.35 | 93.50±0.24 |

**Table S2.** NO_2_^−^ product analysis of Cu(OH)_6_/Co catalyst with various Co content at −0.7 V.

| Co content  (%) | $\text{Y}_{\text{NO}_{\text{2}}^{\text{−}}}$  (μmol h^−1^ mg^−1^) | $\text{F}_{\text{NO}_{\text{2}}^{\text{−}}}$  (%) | $\text{S}_{\text{NO}_{\text{2}}^{\text{−}}}$  (%) |
| --- | --- | --- | --- |
| 0 | 145.83±1.45 | 77.94±4.16 | 96.71±0.10 |
| 1 | 25.84±1.18 | 11.92±0.99 | 39.91±1.24 |
| 2 | 17.50±0.97 | 6.81±0.31 | 23.94±1.09 |
| 10 | 14.14±0.89 | 4.44±0.12 | 16.88±0.60 |
| 20 | 7.88±0.57 | 2.94±0.09 | 10.64±0.49 |
| 50 | 5.40±0.26 | 1.84±0.05 | 7.72±0.45 |
| 70 | 4.50±0.21 | 1.80±0.04 | 7.27±0.58 |
| 90 | 3.57±0.19 | 1.78±0.04 | 7.07±0.41 |
| 100 | 0.32±0.01 | 1.69±0.04 | 6.50±0.24 |

**Table S3.** NH_3_ product analysis of Cu(OH)_6_/Co catalyst after bulk electrolysis at various potentials.

| Potential  (V vs. RHE) | $\text{Y}_{\text{NH}_{\text{3}}}$  (μmol h^−1^ mg^−1^) | $\text{F}_{\text{NH}_{\text{3}}}$  (%) | $\text{S}_{\text{NH}_{\text{3}}}$  (%) |
| --- | --- | --- | --- |
| −0.1 | 0.15±0.01 | 6.14±0.12 | 10.07±0.64 |
| −0.2 | 0.15±0.02 | 3.45±0.09 | 5.56±0.72 |
| −0.3 | 0.37±0.04 | 10.56±0.27 | 8.71±0.93 |
| −0.4 | 12.37±0.97 | 56.09±1.69 | 72.60±1.80 |
| −0.5 | 16.18±1.02 | 78.23±2.01 | 70.01±1.44 |
| −0.6 | 52.55±0.79 | 87.45±2.67 | 89.10±0.48 |
| −0.7 | 64.51±1.57 | 90.01±2.57 | 92.28±0.45 |
| −0.8 | 48.18±1.41 | 65.52±1.91 | 90.02±0.49 |
| −0.9 | 38.23±1.52 | 51.51±1.72 | 88.03±0.52 |

**Table S4.** NO_2_^−^ product analysis of Cu(OH)_6_/Co catalyst after bulk electrolysis at various potentials.

| Potential  (V vs. RHE) | $\text{Y}_{\text{NO}_{\text{2}}^{\text{−}}}$  (μmol h^−1^ mg^−1^) | $\text{F}_{\text{NO}_{\text{2}}^{\text{−}}}$  (%) | $\text{S}_{\text{NO}_{\text{2}}^{\text{−}}}$  (%) |
| --- | --- | --- | --- |
| −0.1 | 1.34±0.03 | 13.74±0.29 | 89.93±0.64 |
| −0.2 | 2.55±0.08 | 15.06±0.35 | 94.44±0.72 |
| −0.3 | 3.88±0.17 | 27.54±0.91 | 91.29±0.93 |
| −0.4 | 4.67±0.21 | 34.26±1.37 | 27.40±1.80 |
| −0.5 | 6.93±0.19 | 8.37±0.91 | 29.99±1.44 |
| −0.6 | 6.43±0.23 | 5.96±0.42 | 10.90±0.48 |
| −0.7 | 5.40±0.26 | 1.84±0.05 | 7.72±0.45 |
| −0.8 | 5.34±0.18 | 1.81±0.03 | 9.98±0.49 |
| −0.9 | 5.20±0.15 | 1.75±0.02 | 11.97±0.52 |

**Table S5.** NH_3_ products analysis of Cu(OH)_6_ catalyst after bulk electrolysis at various potentials.

| Potential  (V vs. RHE) | $\text{Y}_{\text{NH}_{\text{3}}}$  (μmol h^−1^ mg^−1^) | $\text{F}_{\text{NH}_{\text{3}}}$  (%) | $\text{S}_{\text{NH}_{\text{3}}}$  (%) |
| --- | --- | --- | --- |
| −0.1 | 0.01±0.01 | 2.81±0.24 | 1.20±1.19 |
| −0.2 | 0.14±0.02 | 4.39±0.36 | 1.67±0.26 |
| −0.3 | 0.34±0.04 | 5.78±0.31 | 1.51±0.19 |
| −0.4 | 0.56±0.04 | 6.66±0.42 | 1.36±0.11 |
| −0.5 | 0.99±0.06 | 7.83±0.48 | 1.30±0.08 |
| −0.6 | 3.21±0.12 | 11.57±0.97 | 2.59±0.10 |
| −0.7 | 4.96±0.14 | 21.20±1.53 | 3.29±0.10 |
| −0.8 | 19.32±0.37 | 36.75±2.04 | 12.04±0.23 |
| −0.9 | 40.52±0.56 | 65.39±3.73 | 23.16±0.30 |

**Table S6.** NO_2_^−^ products analysis of Cu(OH)_6_ catalysts after bulk electrolysis at various potentials.

| Potential  (V vs. RHE) | $\text{Y}_{\text{NO}_{\text{2}}^{\text{−}}}$  (μmol h^−1^ mg^−1^) | $\text{F}_{\text{NO}_{\text{2}}^{\text{−}}}$  (%) | $\text{S}_{\text{NO}_{\text{2}}^{\text{−}}}$  (%) |
| --- | --- | --- | --- |
| −0.1 | 0.82±0.03 | 23.03±0.24 | 98.80±1.19 |
| −0.2 | 8.22±0.53 | 29.71±1.58 | 98.33±0.26 |
| −0.3 | 22.17±1.28 | 36.89±2.01 | 98.49±0.19 |
| −0.4 | 40.60±1.49 | 42.80±2.34 | 98.64±0.11 |
| −0.5 | 74.88±1.37 | 50.92±2.47 | 98.70±0.08 |
| −0.6 | 120.84±1.29 | 64.39±3.57 | 97.41±0.10 |
| −0.7 | 145.83±1.45 | 77.94±4.16 | 96.71±0.10 |
| −0.8 | 141.17±1.55 | 60.56±3.84 | 87.96±0.23 |
| −0.9 | 134.40±1.23 | 29.54±3.12 | 76.84±0.30 |

**Table S7.** NH_3_ products analysis of Co nanoparticles after bulk electrolysis at various potentials.

| Potential  (V vs. RHE) | $\text{Y}_{\text{NH}_{\text{3}}}$  (μmol h^−1^ mg^−1^) | $\text{F}_{\text{NH}_{\text{3}}}$  (%) | $\text{S}_{\text{NH}_{\text{3}}}$  (%) |
| --- | --- | --- | --- |
| −0.1 | 0.55±0.02 | 85.43±1.25 | 83.33±1.36 |
| −0.2 | 1.04±0.03 | 86.45±1.51 | 85.95±0.79 |
| −0.3 | 1.48±0.02 | 89.06±2.08 | 87.06±1.04 |
| −0.4 | 1.93±0.04 | 81.24±1.68 | 83.19±0.46 |
| −0.5 | 2.38±0.06 | 79.23±1.34 | 79.33±0.67 |
| −0.6 | 3.49±0.10 | 87.64±1.29 | 89.26±0.53 |
| −0.7 | 4.60±0.11 | 90.04±2.35 | 93.50±0.24 |
| −0.8 | 15.74±0.62 | 75.48±2.04 | 95.63±0.20 |
| −0.9 | 27.32±1.11 | 70.22±2.73 | 95.36±0.21 |

**Table S8.** NO_2_^−^ products analysis of Co nanoparticles after bulk electrolysis at various potentials.

| Potential  (V vs. RHE) | $\text{Y}_{\text{NO}_{\text{2}}^{-}}$  (μmol h^−1^ mg^−1^) | $\text{F}_{\text{NO}_{\text{2}}^{-}}$  (%) | $\text{S}_{\text{NO}_{\text{2}}^{-}}$  (%) |
| --- | --- | --- | --- |
| −0.1 | 0.11±0.01 | 1.73±0.04 | 16.67±1.36 |
| −0.2 | 0.17±0.01 | 2.67±0.05 | 14.05±0.79 |
| −0.3 | 0.22±0.02 | 3.25±0.06 | 12.94±1.04 |
| −0.4 | 0.39±0.01 | 4.67±0.08 | 16.81±0.46 |
| −0.5 | 0.62±0.02 | 5.20±0.10 | 20.67±0.67 |
| −0.6 | 0.42±0.02 | 3.60±0.07 | 10.74±0.53 |
| −0.7 | 0.32±0.01 | 1.69±0.04 | 6.50±0.24 |
| −0.8 | 0.72±0.02 | 1.21±0.03 | 4.37±0.20 |
| −0.9 | 1.33±0.03 | 0.86±0.02 | 4.64±0.21 |

**Table S9.** Comparison of the catalytic performance of Cu(OH)_6_/Co with reported catalysts

| **Catalysts** | **Loading**  **(mg cm⁻^2^)** | **Electrode area (cm^2^)** | **Reactor** | **Electrolyte** | **Potential**  **(V vs. RHE)** | **Yield** | **Selectivity (%)** | **FE**  **(%)** | **Reference** |
| --- | --- | --- | --- | --- | --- | --- | --- | --- | --- |
| CuSn(OH)₆/Co | 0.5 | 0.5 × 0.5 CC | H Cell | 0.1 M M Na_2_SO_4_ + 10 mM NaNO_3_  0.1 M M Na_2_SO_4_ + 0.1 M NaNO_3_ | −0.7 | 64.51 μmol h^−1^ mg_Cat_⁻¹  196.13 μmol h^−1^ mg_Cat_⁻¹ | 92.28  94.52 | 90.01  94.52 | This work |
| CoPhz/CNT | 1 | 1 × 1 CP | H Cell | 0.1 M Na_2_SO_4_ + 0.1 M KNO_3_ | −0.65 | 8347.9 µg h^−1^ cm^−2^ | / | 93 | *Angew. Chem. Int. Ed.* **2026**, 65, e24566 |
| Pd⊂QA-Cage^24+^ | / | 1 × 1 CP | H Cell | 0.5 M Na_2_SO_4_ + 0.1 M NaNO_3_ | –0.8 | 25.70 mg h^−1^ mg_Cat_^−1^ | / | 95.44 | *Angew. Chem. Int. Ed.* **2026**, 65, e4155022. |
| Cu_2_-S_1_N_4_/HCS | / | 1 × 1 NF | H Cell | 0.05 M Na_2_SO_4_ + 500 mg L^−1^ NO_3_^−^-N | −0.6 | 11.8 mg h^−1^ cm^−2^ | 80.3% | 93.1 | *Adv. Funct. Mater.* **2026**, 36, e28731 |
| Co-Cu_2_O NRs | / | 1 × 1 | H Cell | 0.5 M Na_2_SO_4_ + 200 mg/L NO_3_^−^ | −0.7 | 9.35 mg h^−1^ cm^−2^ | / | 94.3 | *Adv. Funct. Mater.* **2026**, 36, e75417 |
| Cu-QCS-R | 4 | CC | H Cell | 0.5 M Na_2_SO_4_+0.1 M NaNO_3_ | −0.45 | 0.1 mmol h^−1^ cm^−2^ | / | 95.8 | ***J. Mater. Chem. A*** **2026**, **14**, 13042 |
| (RuZn)/Ni-CN | 0.25 | 1 × 1 CP | H Cell | 0.1 M Na_2_SO_4_ + 0.1 M NaNO_3_ | −0.9 | 10199 µg h^−1^ mg_Cat_^−1^ | / | 84 | *Small* **2026**, 22, e12253 |
| TTA-TPH-CuCo | / | 1 × 1 CP | H Cell | 0.5 M K_2_SO_4_ + 0.3 M KNO_3_ | −0.75 | 20.8 mg·h^−1^·cm^−2^ | / | 92.16 | *Angew. Chem. Int. Ed.* **2025**, 64, e202507956 |
| W-CCNs | 0.1 | 1 × 1 | H Cell | 1 M Na_2_SO_4_ + 0.1 M NaNO_3_ | −0.9 | 75.3 mg·h^−1^·cm^−2^ | / | 70.0 | *Adv. Mater.* **2025**, 37, 2417623 |
| Cu-CoP | / | / | H Cell | 0.1 M K_2_SO_4_ + 0.01 M NaNO_3_ | −1.0 | 7.65 mg h^−1^ cm^−2^ | / | 85.1 | *Adv. Sci.***2025**, 12, 2416386. |
| Mo_2_C@BNHCFs-2 | / | 1 × 1 CP | H Cell | 0.5 M Na_2_SO_4_ + 0.05 M NaNO_3_ | −1.1 | 6487.43 μg h^−1^ mg_cat_^−1^ | / | 74.5 | *ChemSusChem* **2025**, 18, e202500059 |
| Co-bpta-btc | / | CC | H Cell | 0.05 M Na_2_SO_4_ + 0.1 M NO_3_^−^ | −1.0 | 10.3 mg h^−1^ mg_cat_^−1^ | / | 83.3 | *Inorg. Chem.* **2025**, 64, 27, 14012 |
| MnFeCoNiCu HEA | 0.1 | CP | H Cell | 0.5 M Na_2_SO_4_ + 0.1 M NaNO_3_ | −0.6 | 10.2 g h^−1^ mg_cat_^−1^ | / | 94.5 | *Adv. Mater.* **2025**, 37, 2415739 |
| MoS_2_@V_2_C-0.75 | / | CP | H Cell | 0.5 M Na_2_SO_4_ + 0.1 M NaNO_3_ | −0.9 | 708 μg·h^−1^·mg^−1^ | 84 | 80 | *Nano Res.* **2025**, 18, 94907521 |
| NiCoO_2_@Cu | / | / | H Cell | 0.1 M Na_2_SO_4_ + 0.1 M NaNO_3_ | −0.9 V | 5940.73 μg h^–1^ cm^–2^ | / | 94.2 | *ACS Appl. Mater. Interfaces* **2024**, 16, 11431 |
| Fe-HESA NCs | 0.05 | 1.5  × 1 GCE | H Cell | 0.5 M Na_2_SO_4_ + 100 mM NaNO_3_ | −0.6 | 81.4 mg h^−1^ mg^−1^ | / | 93.4 | *Nat. Commun.* **2024**, 15, 6932 |
| FL-Ag/HEA | / | / | H Cell | 0.5 M K_2_SO_4_ + 0.1 M KNO_3_ | – 0.57 | 2.45 mmol h^–1^ mg_cat._^–1^ | / | 92.7 | *Nat. Commun.* **2024**, 15, 9020 |
| Fe-Cu | 0.2 | 1 × 1 CP | H Cell | 0.5 M Na₂SO₄ + 100 ppm NO_3_^−^-N | −0.9 | 323.1 mmol h^–1^ mg_Cat_^–1^ | / | 93.4 | *J. Mater. Chem. A* **2024**,12, 11109 |
| cd-Pd/Cu_2_O NP | / | CP | / | 0.5 M Na₂SO₄ + 50 mM PBS (pH 7) + 100 mM NaNO_3_ | −0.9 | 2.79 mmol h^−1^ mg_cat_^−1^ | / | 95.8 | *Chem. Catal.* **2024**, 4, 101029 |
| Co+Bi@Cu NW | / | / | H Cell | 0.1 M Na_2_SO_4_ + 50 mM NaNO_3_ | −0.6 V | 1858.2 µg h^−1^ cm^−2^ | / | 99.51 | *Adv. Mater.* **2023**, 35, 2306633 |
| Zn–CuO NAs | / | / | H Cell | 0.1 M Na_2_SO_4_ + 0.01 M NaNO_3_ | −0.7 | 945.1 μg h^–1^ cm^–2^ | / | 95.6 | *ACS Appl. Mater. Interfaces* **2023**, 15, 5172 |
| Cu₂O/NiO | / | / | H Cell | 0.5 M Na_2_SO_4_ + 200 ppm NaNO_3_ | −0.2 | 0.21 mmol h^−1^ cm^−2^ | 88.5 | 95.6 | *Adv. Funct. Mater.* **2023**, 33, 2303803 |
| Zn/Cu-2.3 | 0.14 | 0.5 cm^2^ CP | H Cell | 0.5 M K_2_SO_4_ +0.1 M KNO_3_ | −0.55 | 5.8 mmol h^–1^ mg_Cat_^–1^ | / | 98.4 | *Angew. Chem. Int. Ed.* **2023**, 62, e202307952 |
| RuFe-FeNC | / | 1 × 1 CP | H Cell | 0.5 M K_2_SO_4_ + 0.1 M KNO_3_ | −1.4 | 118.8 mg h^−1^ mg^−1^ | 90.3 | 92.2 | *Adv. Energy Mater.* **2023**, 13, 2301409 |
| Cu/MnOx | / | / | / | 0.1 M K_2_SO_4_ + 100 mM KNO_3_ | −0.6 | 29.3 mg h^−1^ mg_cat_^−1^ | / | 86.2 | *Small* **2023**, 19, 2207661 |
| Co_3_O_4_/Co | / | CC | H CellH Cell | 0.5 M Na₂SO₄ + 0.1 M NaNO₃ | −0.8 | 4.43 mg h^−1^ cm^−2^ | / | 88.7 | *Chem. Eng. J.* **2023**, 461, 141960 |
| FOSP-Cu | / | / | H Cell | 0.1 M Na_2_SO_4_ + 100 ppm NO_3_^−^-N | −0.60 | 101.4 μmol·h⁻¹·cm⁻² | / | 93.91 | *Nano Energy* **2022**, 97, 107124 |
| Cu–Co_3_O_4_ | / | / | H Cell | 0.1 M Na_2_SO_4_ + 500 ppm NaNO_3_ | −0.6 | 36.71 μmol h^–1^ mg_Cat_^–1^ | / | 86.5 | *ACS Appl. Mater. Interfaces* **2022**, 14, 35477 |
| Pd-Cu_2_O  CEO | 1 | 1 × 1 CP | H Cell | 0.5 M Na₂SO₄ + 50 ppm NO_3_^−^-N | −1.3 | 925.11 µg h^−1^ mg_cat_^−1^ | 95.31 | 96.56 | *Appl. Catal. B Environ.* **2022**, 306. 121094 |
| NbO*_x_* | / | 1 × 1 CP | H Cell | 0.5 M K_2_SO_4_ + 1000 ppm NO_3_^−^ | − 1.15 | 55.0 μg·h^−1^·mg_cat_^−1^ | / | 94.5 | *Green Chem.* **2022**, 24, 1090 |
| Fe SAC | 0.4 | 1.5  × 1 GCE | H Cell | 0.1 M K_2_SO_4_  + 0.50 MNaNO_3_ | −0.66 | 5245 μg h^−1^ mg_cat_^−1^ | / | 75 | *Nat. Commun.* **2021**, 12, 2870 |
| Cu/Cu_2_O NWAs | / | 1  × 1 Cu mesh | H Cell | Na_2_SO_4_ + 0.5 M 200 ppm NO_3_^−^-N | −0.85 | 0.2449 mmol h^−1^ cm^−2^ | 81.2 | 95.8 | *Angew. Chem. Int. Ed.* **2020**, 59, 5350 |

**Note:** CP represents Carbon paper, CC represents carbon cloth, NF represents nickel foam, and GCE represents glassy carbon electrode.
